# Photoswitchable Active Esters for the Control of Amide Bond Formation with Light

**DOI:** 10.1021/jacsau.5c00930

**Published:** 2025-10-03

**Authors:** Marc Villabona, Arnau Marco, Rosa María Sebastián, Christopher Barner-Kowollik, Gonzalo Guirado, Jordi Hernando

**Affiliations:** † Departament de Química, 16719 Universitat Autònoma de Barcelona (UAB), Edifici C/n, Campus UAB, Cerdanyola del Vallès 08193, Spain; ‡ Institute of Nanotechnology (INT) and Institute of Functional Interfaces (IFG), Karlsruhe Institute of Technology (KIT), Hermann-von-Helmholtz-Platz 1, Eggenstein-Leopoldshafen 76344, Germany; § School of Chemistry and Physics, Centre for Materials Science, 1969 Queensland University of Technology (QUT), 2 George Street, Brisbane 4000, Queensland, Australia

**Keywords:** amidation, photoswitches, diarylethenes, light-controlled reactivity, active esters

## Abstract

Active esters are among the most utilized reagents for (bio)­materials functionalization via amide bond formation. To endow this type of ligation processes with spatiotemporal precision, we herein report the development of photoswitchable active esters, which toggle between poorly and highly reactive states upon irradiation with UV and visible light. Specifically, a dithienylethene photoswitch was introduced within the structure of common *p*-nitrophenyl active esters, whose electronic changes upon reversible open-closed photoisomerization turn on and off the activating effect of the *p*-nitro substituent on amidation reactivity. As a result, efficient light-induced modulation of amide bond formation kinetics was accomplished with these compounds, with their closed isomer exhibiting up to 24-fold enhancement of aminolysis rate with amines relative to the open state. This behavior was exploited to reach reversible light-control of illustrative examples of amidation-based ligation processes: dye labeling, polymer gelation and polymer thin film patterning, which was selectively triggered by illumination at 365 nm and inhibited by irradiation at 625 nm. These results demonstrate the potential of photoswitchable active esters to provide enhanced spatiotemporal control to the functionalization and manipulation of molecules and materials.

## Graphical abstract

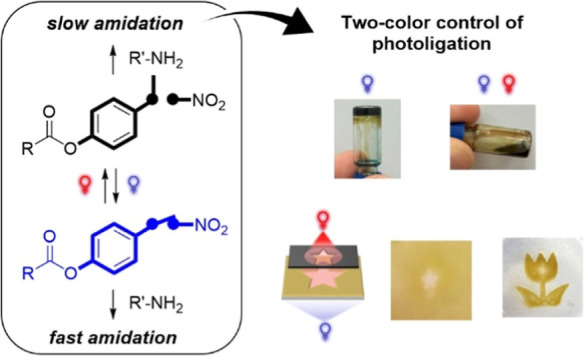

## 1 Introduction

Amide bond formation, a pivotal transformation in organic synthetic chemistry,^1^ is one of the primary strategies used for the functionalization of biomolecules,^2^ polymer networks,^3^ nanostructures,^4^ and surfaces and thin films.^5^ For many of these applications, on-demand control of amide coupling with light would be highly desirable, as photoinduced reactions allow chemical ligation with spatiotemporal resolution,^6^ and therefore have become powerful tools for bioconjugation,^7^ surface patterning^8^ advanced printing,^9^ and the postsynthetic modification of materials.^10^ However, although the development of light-mediated amidation reactions has rapidly evolved in the last ten years with the emergence of photoredox catalysis, attention has so far been focused on establishing synthetic procedures that enable amide bond formation at milder conditions than classical methods,^11^ while their use for photoligation in materials science or biological substrates has been scarcely considered.^12^ In addition, all these precedents share a common feature that compromises the spatiotemporal precision with which photoinduced amidation can be accomplished: reactivity is triggered by irradiation with a single source of light, which (1) cannot preclude photoactivated reagents to diffuse and react outside of the irradiated region, and (2) does not enable reaction confinement on the nanoscale due to the diffraction limit imposed by photoexcitation with far-field optics.^13^

One of the methodologies proposed to surpass these limitations is the antagonistic control of photoligation processes with two orthogonal wavelengths, where one color of light is used to trigger a covalent reaction and another to suppress it.^6b,6c,14^ By developing photoswitchable reagents that reversibly toggle between reactive and nonreactive states upon photoisomerization, this strategy has been successfully applied to lithography and nanofabrication,^15^ polymer manipulation,^16^ and bioconjugation.^17^ In most of these cases, photoswitching is utilized to regulate cycloaddition reactions with two colors of light,^15,16a,16b,17,18b^ though its use to reversibly modulate nucleophile addition to aldehydes and ketones,^16c,16e,16h,18a^ Michael acceptors,^16f,16g^ and boronic esters^16d^ has also been reported. In contrast, its application to light-control amide bond formation has not been considered yet, except for a photoswitchable supramolecular receptor particularly designed to accelerate the amidation reaction between two specific adenosine-containing substrates.^19^ Therefore, herein we aim to expand the toolbox of dual-wavelength controlled photoligation processes to amidation processes, thereby granting enhanced spatiotemporal precision to one of the most popular approaches used for chemical functionalization and manipulation.

Our strategy for photoswitchable amidation is based on the well-known chemistry of active esters, which are among the most widely used substrates for amide preparation.^1b–1d,20^ This is the case for *p*-nitrophenyl (PNP) esters, which constitute one of the first examples of active esters described.^21^ As their electron-withdrawing *p*-nitro substituent enhances the electrophilicity of the carbonyl moiety and lowers the basicity of the *p*-nitrophenolate leaving group, PNP esters undergo fast aminolysis, in contrast to the esters of electron-richer phenols and common alkyl esters (Figure 1a).^21^ Herein, we reversibly control amide bond formation with light by introducing photoswitchable motifs into PNP esters, whose photoisomerization could turn on and off the electronic effect of the *p*-nitro group on reactivity. Specifically, we selected dithienylethenes (DTE) as photoswitching units, because their photoconversion between ring-open (o) and ring-closed (c) states modulates the electronic communication between the external substituents of their thiophene rings.^22^ This feature has already been exploited to optically control the reactivity of several DTE-appended groups^16c,18b^ and, very recently, we have demonstrated that it allows photomodulating the acidity of phenols.^23^ Thus, by tethering phenol and nitro groups through a DTE photoswitch, the phenol acidity was modified ca. 5 p*K*_a_ units as a result of the change in electronic conjugation that occurs upon photoisomerization. Herein, we hypothesize that the acylation of such nitro-DTE-phenol conjugates could afford photoswitchable phenyl esters (DTE-PNP) with light-regulated amidation reactivity, which may then be applied to conduct two-color controlled photoligation processes. As shown in Figure 1b, these compounds should exhibit slow aminolysis rates in the open state where the phenoxy moiety is not conjugated to the nitro group but to a thiophene ring, which should impart negligible electronic effects according to its Hammett σ-*para* substituent constant (σ_p_ = 0.05 ^24^). In contrast, amidation reactivity should be activated upon photocyclization by enabling electronic communication between the phenoxy group and the electron-withdrawing nitro substituent (σ_p_ = 0.78 ^24^) through the planar conjugated backbone of the closed DTE isomer, thereby mimicking the structure of active PNP esters.

**Figure 1:**
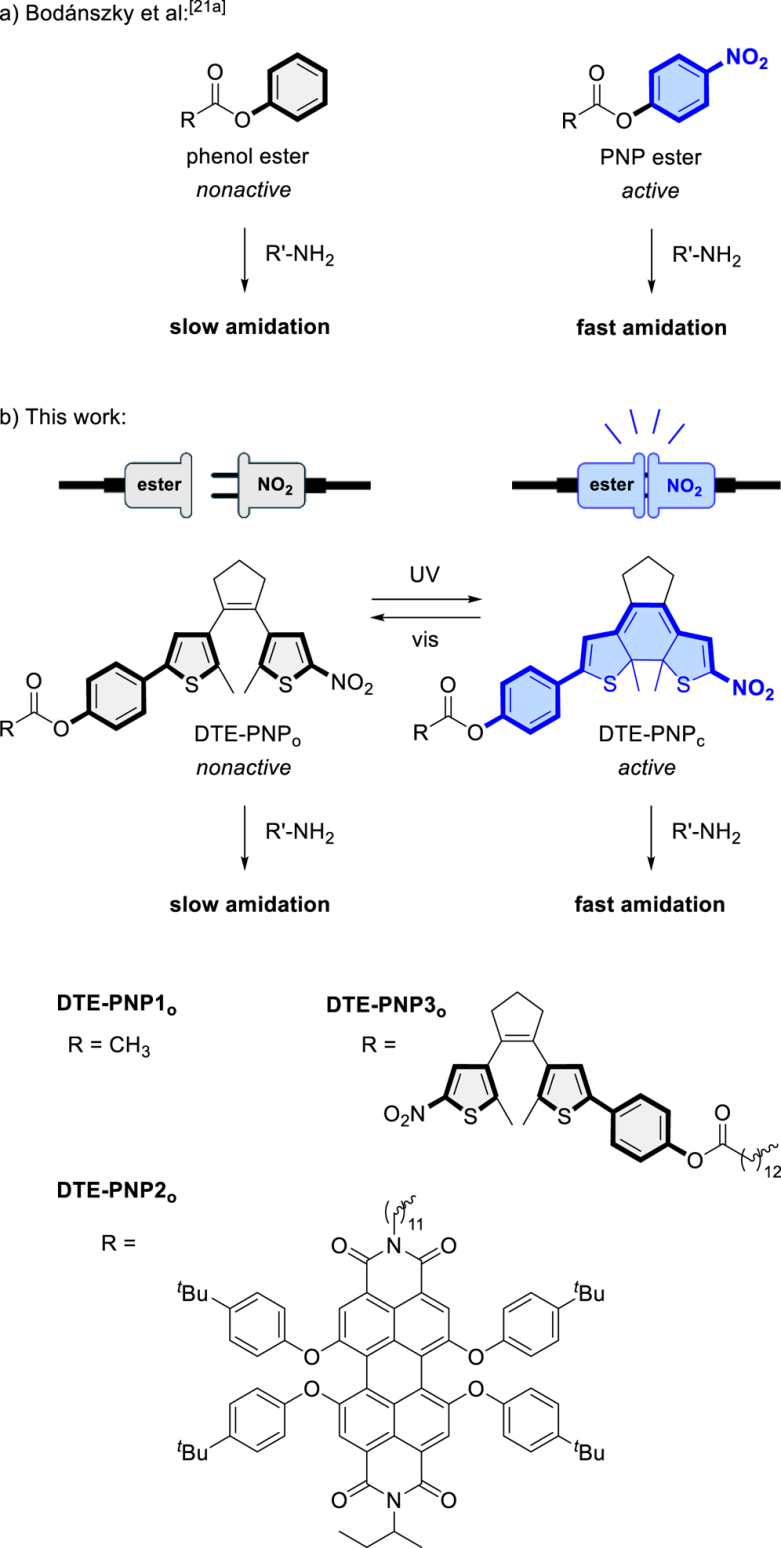
(a) Difference in aminolysis reactivity described for esters of electron-deficient and electron-richer phenols (e.g., *p*-nitrophenyl and phenyl esters^21a^). (b) Strategy devised in this work for light-controlling amidation reactions using photoswitchable active esters (DTE-PNP), where a photoisomerizable dithienylethene unit is used to switch on and off the electronic communication between phenyl esters and an activating nitro group. This strategy has been applied to model (**DTE-PNP1**) and functional (**DTE-PNP2**, **DTE-PNP3**) photoswitchable active esters.

## 2 Results and Discussion

### 2.1 Light-Control of Amidation with Photoswitchable Active Esters

To validate our approach for the light-control of amidation, we initially investigated the reactivity of a model DTE-PNP compound in solution. For simplicity, we selected the acetate ester **DTE-PNP1** as a benchmark system, which was prepared by direct acylation with acetyl chloride of the photoswitchable phenol **DTE-NO**_**2**_ previously synthesized by us^23^ (Figure 2a). Notably, the resulting ester preserved the photoswitching properties of **DTE-NO**_**2**_. In solvents of low and intermediate polarity (e.g., toluene, CHCl_3_ and THF), the open isomer **DTE-PNP1**_**o**_ efficiently photoisomerized to the closed state **DTE-PNP1**_**c**_ upon irradiation at 365 nm, with photocyclization quantum yields (Φ_o‑c_) exceeding 0.15 and conversions around 95%, which may be further optimized by a detailed analysis via a photochemical action plot to map wavelength dependent quantum yields that typically do not align with molar absorptivity.^25^ Subsequent illumination at 625 nm promoted quantitative back-photoisomerization to the initial open isomer with lower quantum yields (Φ_c‑o_ ∼ 0.007–0.009), which enabled repetitive photoswitching of **DTE-PNP1** with minor photodegradation under sequential UV- and red-light irradiation (Figure 2b–c, Table S1 and Figures S1–S3). In contrast, thermal back-isomerization was not observed for **DTE-PNP1**_**c**_ at room temperature, which remained stable in the dark for at least 48 h (Figure S4). As already reported for push–pull substituted DTE compounds,^23,26^ the photochemical performance of **DTE-PNP1** slightly declined in solvents of higher polarity such as acetonitrile and DMSO (Φ_o‑c_ < 0.06), although good ring-closing photoconversions could still be achieved under UV irradiation (>70%), while the red-light induced photocycloreversion reaction remained quantitative in these media (Table S1, Figures S2 and S3).

**Figure 2:**
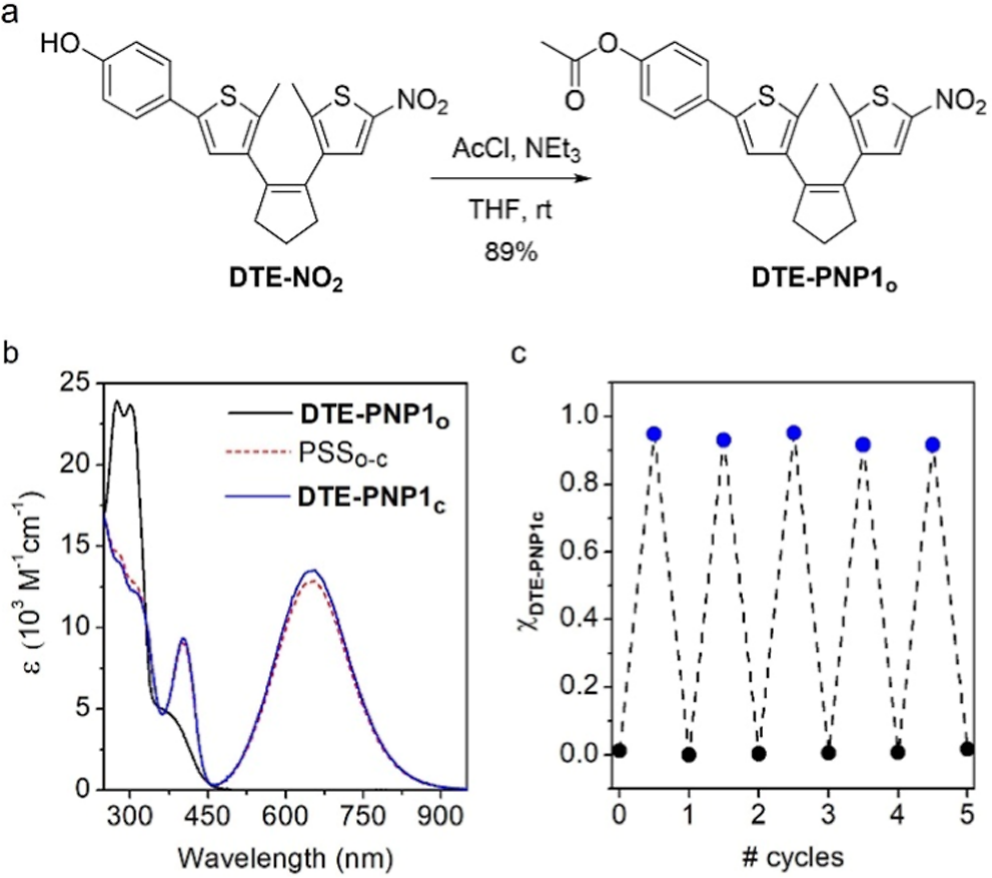
(a) Synthesis of photoswitchable active ester **DTE-PNP1**. (b) UV–vis absorption spectra of the open and closed isomers of **DTE-PNP1** in CHCl_3_, as well as of the photostationary state mixture obtained upon irradiation of **DTE-PNP1**_**o**_ at 365 nm (PSS_o‑c_). (c) Variation of the molar fraction of the closed isomer **DTE-PNP1**_**c**_ upon consecutive cycles of irradiation of a CHCl_3_ solution of **DTE-PNP1** at 365 nm (for photocyclization) and 625 nm (for photocycloreversion).

To investigate the modulation of amidation reactivity for the two isomers of **DTE-PNP1**, we monitored their reaction with a model primary alkylamine (1-dodecylamine) in aprotic solvents of varying polarity (CHCl_3_, THF and acetonitrile) at room temperature by ^1^H NMR spectroscopy (Figures 3a and S5). Specifically, separate aminolysis reactions were conducted for pure **DTE-PNP1**_**o**_ and UV-induced photostationary mixtures enriched with **DTE-PNP1**_**c**_ (>80%), using a 20-fold excess of amine to make amide bond formation follow pseudo-first order kinetics (*c*_DTE‑PNP1_ = 5.0 mM, *c*_amine_ = 100 mM). However, only the apparent pseudo-first order rate coefficients are reported herein (*k* ^obs^), as their dependence on amine concentration is often not linear due to self-catalytic effects and may vary with the nature of the solvent and substrate.^27^ For comparison purposes, similar measurements were performed for the acetate of *p*-nitrophenol (**PNP1**) as a reference active ester, using UV–vis absorption spectroscopy to monitor reaction kinetics in those solvents where amidation took place very fast (<20 min) (Figures 3a and S6). Attempts to expand these experiments to aqueous media (such as acetonitrile/water mixtures) were unsuccessful due to poor **DTE-PNP1** and **PNP1** solubility.

**Figure 3:**
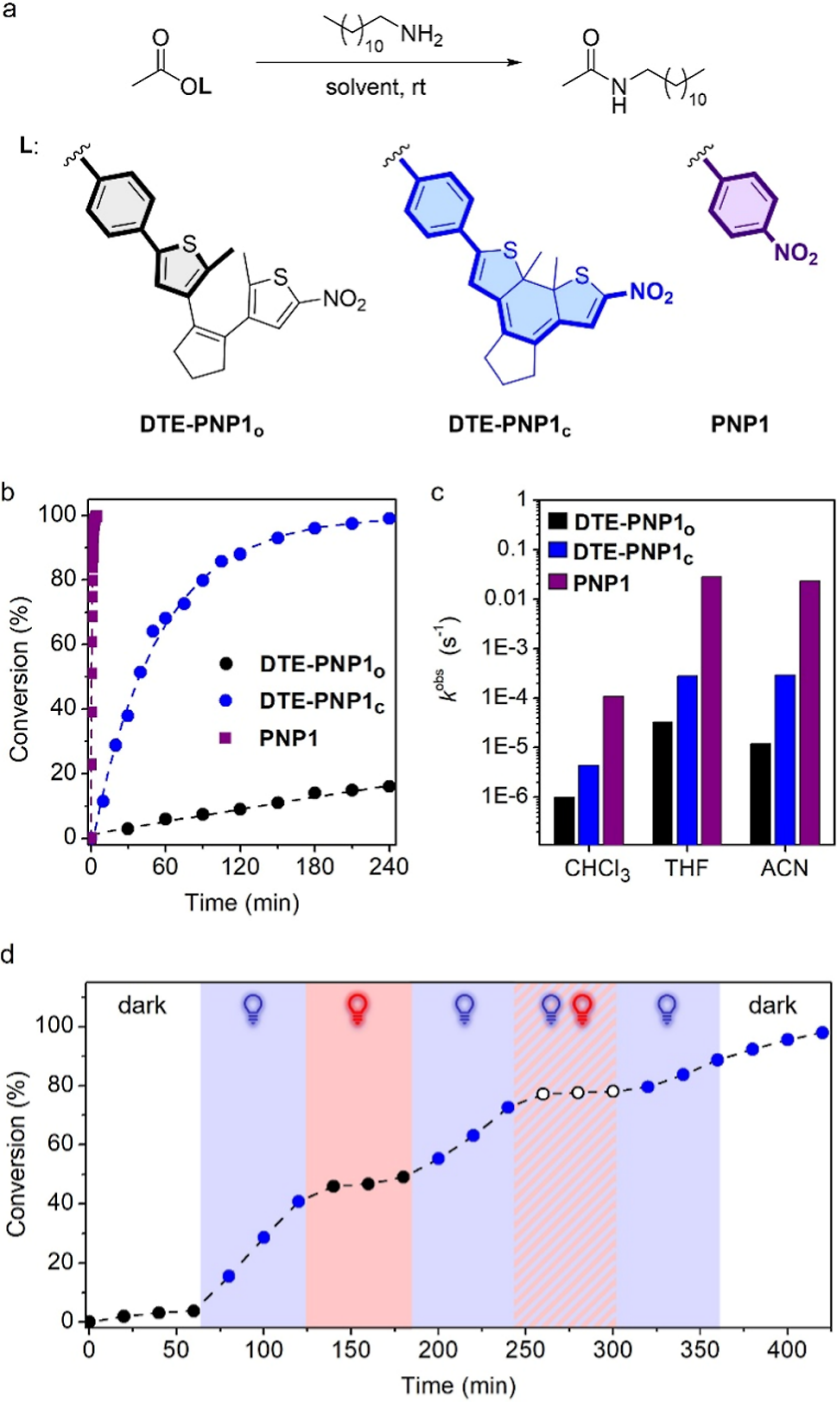
(a) Aminolysis reaction between **DTE-PNP1** (*c* = 5.0 × 10^–3^ M) or **PNP1** (*c* = 5.0 × 10^–3^ M) with 1-docecylamine (*c* = 0.10 M) at room temperature. (b) Evolution of aminolysis conversion with reaction time for **DTE-PNP1**_**o**_, **DTE-PNP1**_**c**_ and **PNP1** in acetonitrile at room temperature. Data points are experimental data obtained from ^1^H NMR or UV–vis absorption spectra (see the Supporting Information for further details), while lines are fits to a pseudo-first order kinetic model (fit error <5%). Full conversion for **DTE-PNP1**_**o**_ was only observed after 6 days. (c) Pseudo-first order rate constants for the aminolysis reaction of **DTE-PNP1**_**o**_, **DTE-PNP1**_**c**_ and **PNP1** in chloroform, THF and acetonitrile at room temperature. (d) Evolution of aminolysis conversion with reaction time for **DTE-PNP1**_**o**_ in acetonitrile at room temperature when subjected to different sequential illumination conditions: (i) in the dark (0–60 min); (ii) irradiation at λ_max_ = 365 nm and 0.017 mW cm^–2^ (60–120 min); (iii) irradiation at λ_max_ = 625 nm and 400 mW cm^–2^ (120–180 min); (iv) irradiation at λ_max_ = 365 nm and 0.017 mW cm^–2^ (180–240 min); (v) combined irradiation at λ_max_ = 365 nm (0.017 mW cm^–2^) and λ_max_ = 625 nm (400 mW cm^–2^) (240–300 min); (vi) irradiation at λ_max_ = 365 nm and 0.017 mW cm^–2^ (300–360 min); and (vii) in the dark (360–420 min). Data obtained from ^1^H NMR spectra (see the Supporting Information for further details).

When analyzing the amidation rate coefficients determined for **DTE-PNP1** and **PNP1** in different solvents, several conclusions can be drawn (Figure 3b,c, Table 1 and Figure S7). For these compounds, higher reactivity was observed in THF and acetonitrile relative to the less polar solvent CHCl_3_, probably due to the enhanced stabilization of the polar tetrahedral intermediate generated in the first step of the amidation process by amine addition to the carbonyl group.^27^ In addition, larger rate coefficients were measured for **PNP1** than for the two states of **DTE-PNP1** in all solvents. We ascribe this result to the larger distance between the carbonyl and nitro groups in **DTE-PNP1**_**c**_ relative to **PNP1**, as rotation of the bond that links the phenyl ester and DTE_c_ fragments may attenuate the activating effect on amidation reactivity due to decreased conjugation efficiency. More importantly, we registered a clear variation in amide bond formation kinetics for the two states of **DTE-PNP1**, with the closed isomer **DTE-PNP1**_**c**_ exhibiting larger aminolysis coefficients in all analyzed solvents (*k*_DTE‑PNP1c_^obs^/*k*_DTE‑PNP1o_^obs^ > 4). This effect was found to develop with increasing solvent polarity, leading to a high reactivity amplification factor between the closed and open isomers of **DTE-PNP1** in acetonitrile: *k*_DTE‑PNP1c_^obs^/*k*_DTE‑PNP1o_^obs^ = 24, which is of the same order as previously reported for photoswitchable imine formation using a related DTE-based strategy.^16c^ As a result, the aminolysis reaction of **DTE-PNP1**_**c**_ was completed in only 4 h at room temperature, while only ca. 15% of **DTE-PNP1**_**o**_ was consumed after this reaction time. Interestingly, similar effects on reactivity modulation were observed when 1-dodecylamine was replaced by a secondary amine such as dihexylamine (*k*_DTE‑PNP1c_^obs^/*k*_DTE‑PNP1o_^obs^ = 39 in acetonitrile, Figure S8). Overall, these results validate our molecular design and demonstrate that switching on and off the electronic communication between phenol esters and nitro groups through a photoisomerizable DTE spacer allows modulation of amidation reactivity.

**Table 1:** Kinetics of the Aminolysis Reaction of the Esters Prepared with 1-Dodecylamine at Room Temperature^a^ ^,^ ^b^

compounds	solvent	*k*_DTE‑PNPo_^obs^ ^c^ (s^–1^)	*k*_DTE‑PNPc_^obs^ ^d^ (s^–1^)	*k*_PNP_^obs^ ^e^ (s^–1^)	*k*_DTE‑PNPc_^obs^/*k*_DTE‑PNPo_^obs^	*k*_PNP_^obs^/*k*_DTE‑PNPc_^obs^
DTE-PNP1 and PNP1	CHCl_3_	1.0 × 10^–6^	4.4 × 10^–6^	1.1 × 10^–4^	4.4	25
	THF	3.3 × 10^–5^	2.8 × 10^–4^	2.8 × 10^–2^	8.4	100
	acetonitrile	1.2 × 10^–5^	2.9 × 10^–4^	2.3 × 10^–2^	24	79
DTE-PNP2 and PNP2	CHCl_3_	8.0 × 10^–7^	6.0 × 10^–6^	2.8 × 10^–5^	7.5	4.7
	THF	7.4 × 10^–6^	1.0 × 10^–4^	1.1 × 10^–2^	14	110
DTE-PNP3 and PNP3^f^	CHCl_3_	1.3 × 10^–7^	1.1 × 10^–6^	3.6 × 10^–5^	8.5	33

a *c*_amine_ = 0.10 M; *c*_ester_ = 5.0 × 10^–3^ M, except for the experiments with **DTE-PNP3** where *c*_DTE‑PNP3_ = 2.5 × 10^–3^ M to make the initial amount of ester groups be 5.0 × 10^–3^ M.

b Kinetics investigated by ^1^H NMR, except for **PNP1** in THF and acetonitrile and **PNP2** in THF, where UV–vis absorption spectroscopy was used.

c Pseudo-first order rate constant for the open isomer of DTE-PNP.

d Pseudo-first order rate constant for the closed isomer of DTE-PNP.

e Pseudo-first order rate constant for the model active ester PNP.

f Rate constants are given per ester group in the dimers.

In light of the large variation of aminolysis rates found for the two states of **DTE-PNP1** in acetonitrile, we explored the two-color control of amide bond formation in situ using this compound as a photoswitchable active ester; i.e., whether amidation could be triggered by UV-induced photocyclization to the more reactive **DTE-PNP1**_**c**_ isomer and inhibited by irradiation with red light to recover the less active **DTE-PNP1**_**o**_ state through back-photoisomerization. To do so, the aminolysis of **DTE-PNP1**_**o**_ with 1-dodecylamine in acetonitrile and at room temperature was monitored by ^1^H NMR spectroscopy while applying a sequence of different illumination conditions (Figure 3d): (1) no irradiation, where very low reactivity was observed for the initial nonactive ester **DTE-PNP1**_**o**_ (*t*_irr_ = 0–60 min); (2) UV illumination at λ_max_ = 365 nm and 0.017 mW cm^–2^, which led to photoconversion to active **DTE-PNP1**_**c**_ and, consequently, fast aminolysis reaction (*t*_irr_ = 60–120 min and *t*_irr_ = 180–240 min); (3) red-light irradiation at λ_max_ = 625 nm and 400 mW cm^–2^, which photoisomerized **DTE-PNP1** back to the less reactive open isomer (*t*_irr_ = 120–180 min); and (4) combined UV (λ_max_ = 365 nm, 0.017 mW cm^–2^) and red irradiation (λ_max_ = 625 nm, 400 mW cm^–2^), where the use of high visible-to-UV light intensity ratio allowed displacing the photostationary equilibrium toward **DTE-PNP1**_**o**_ and, therefore, dramatically slowing amide bond formation (*t*_irr_ = 240–300 min). When the red illumination source was switched off while preserving UV irradiation, **DTE-PNP1**_**c**_ was reformed and the amidation reaction was reactivated (*t*_irr_ = 300–360 min) and subsequently preserved in the dark (*t*_irr_ = 360–420 min), thereby demonstrating that the changes in reactivity observed were not influenced by additional heating effects that could be caused by irradiation. More importantly, the antagonistic response on amidation rate that was achieved under concomitant UV and red light photoexcitation could be exploited to confine the aminolysis process spatially, which we investigated by analyzing the spatial profile of the more reactive **DTE-PNP1**_**c**_ species upon patterned illumination in liquid solution (Figure S9). Thus, while local, single irradiation at λ_max_ = 365 nm resulted in widespread distribution of **DTE-PNP1c** molecules throughout the sample due to diffusion, complementary photoexcitation at λ_max_ = 625 nm with high power allowed their selective localization within the volume element illuminated with UV light. Hence, all these results demonstrate that our model DTE-based photoswitchable active ester enables two-color control of amidation.

### 2.2 Light-Modulation of Amidation with Functional Photoswitchable Active Esters

To explore the potential of our methodology to photomodulate amide bond formation, we prepared a DTE-based photoswitchable active ester bearing a fluorescent moiety that could be transferred to amino-terminated molecular platforms under dual-wavelength control; i.e., an illustrative example of functional DTE-PNP compounds (**DTE-PNP2**) (Figure 4a). For this purpose, a perylene diimide (PDI) fluorophore was selected because of its excellent photophysical properties and facile chemical derivatization.^28^ In addition, the PDI emitter chosen shows low or no absorption at the 365 nm (ε_PDI_ ∼ 0.5 ε_DTEo_) and 625 nm (ε_PDI_ ∼ 0) wavelengths required for DTE photoisomerization and, therefore, it should not significantly interfere with the photocyclization and photocycloreversion reactions of **DTE-PNP2** (Figure S10a). It must be noted that the synthesis of **DTE-PNP2**—and of any other analogous photoswitchable active ester—is straightforward, as it just requires coupling phenol **DTE-NO**_**2**_ to a carboxylated derivative of the functional unit of interest (Scheme S1).

**Figure 4:**
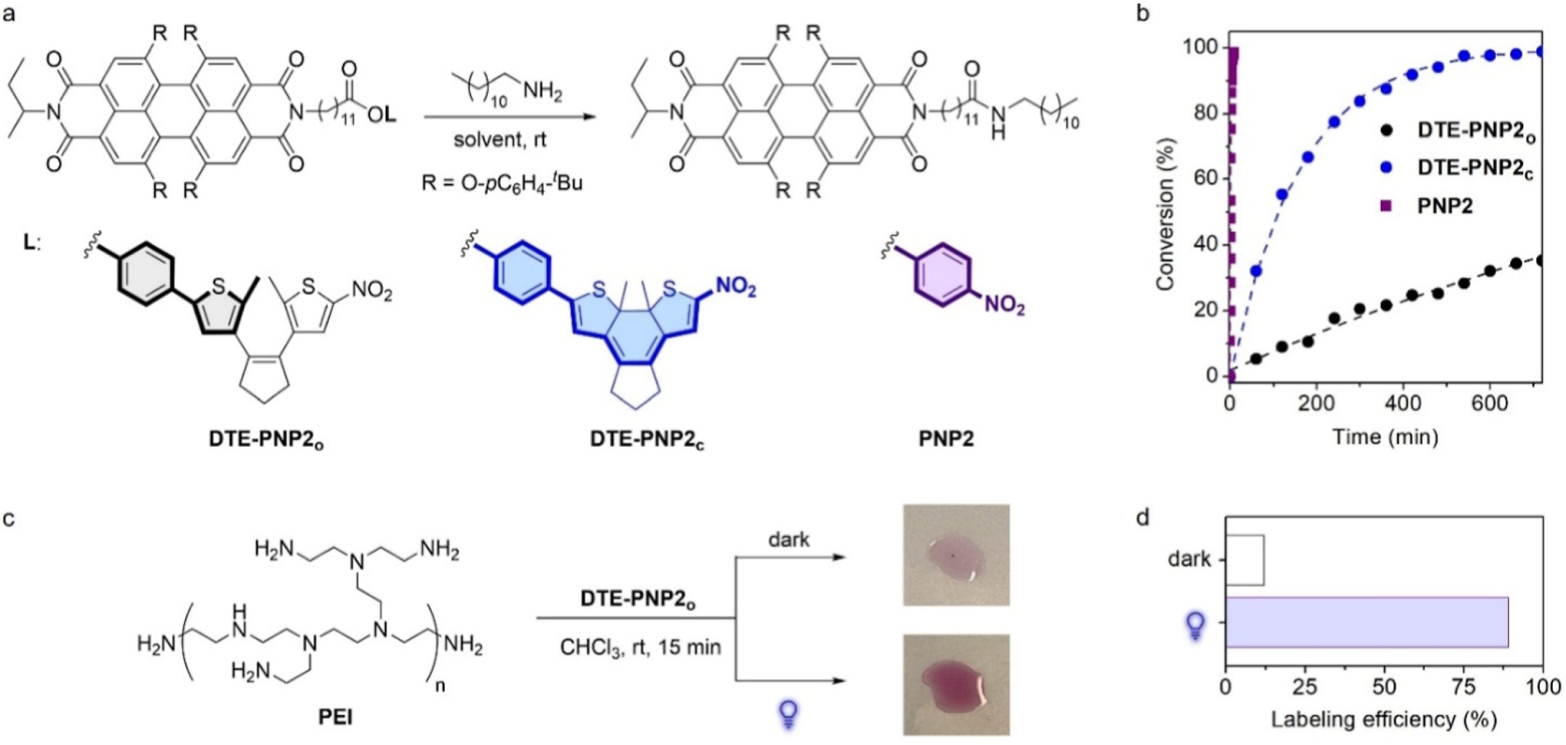
(a) Aminolysis reaction between **DTE-PNP2** (*c* = 5.0 × 10^–3^ M) or **PNP2** (*c* = 5.0 × 10^–3^ M) with 1-docecylamine (*c* = 0.10 M) at room temperature. (b) Evolution of aminolysis conversion with reaction time for **DTE-PNP2**_**o**_, **DTE-PNP2**_**c**_ and **PNP2** in THF at room temperature. Data points are experimental data obtained from ^1^H NMR or UV–vis absorption spectra (see the Supporting Information for further details), while lines are fits to a pseudo-first order kinetic model (fit error <5%). Full conversion was observed for **DTE-PNP2**_**o**_ after 10 days. (c) Photomodulation of **PEI** functionalization with PDI fluorophores using **DTE-PNP2**. Images show the color of the resulting polymer solutions after reaction with **DTE-PNP2**_**o**_ at two different illumination conditions (top) in the dark; and (bottom) after UV irradiation (λ_max_ = 365 nm and 10 mW cm^–2^). (d) PDI labeling efficiency achieved for **PEI** at these two different irradiation conditions according to ^1^H NMR measurements.

Because of its poor solubility in polar organic solvents, the photoswitching behavior of **DTE-PNP2** was only investigated in CHCl_3_ and THF. In a similar fashion to **DTE-PNP1**, irradiation of the open isomer **DTE-PNP2**_**o**_ at 365 nm in these solvents efficiently photoproduced the closed state **DTE-PNP2**_**c**_ (>78% conversion, Φ_o‑c_ = 0.04–0.07), a process that could be quantitatively reverted upon red-light illumination (Φ_c‑o_ ∼ 0.01) and repeated for several photoswitching cycles with good fatigue resistance (Table S2 and Figure S10). In addition, the introduction of a PDI unit in **DTE-PNP2** led to emission detection for both its open and closed states, though with different fluorescence quantum yields (Φ_f,**DTE‑PNP2o**_ = 0.80 and Φ_f,**DTE‑PNP2c**_ = 0.19 in THF) (Table S2 and Figure S11). As the PDI emission spectrum overlaps with the absorbance of the DTE closed isomer, emission quenching in **DTE-PNP2**_**c**_ could be ascribed to resonance energy transfer between these moieties, as previously measured for another PDI-DTE dyad developed by us.^29^ Importantly, such a quenching effect must be inhibited upon **DTE-PNP2**_**c**_ aminolysis and removal of the **DTE-NO**_**2c**_ byproduct, thus allowing the transfer of a DTE-free, bright PDI emitter to the desired amino substrate; for instance, the amide coupling product formed by reaction between **DTE-PNP2**_**c**_ and 12-dodecylamine exhibited intense fluorescence in solution (Φ_f_ = 0.95 in THF).

Aminolysis with 12-dodecylamine was also employed as the benchmark system to assess the modulation of amidation reactivity between the two isomers of **DTE-PNP2** at room temperature, utilizing equivalent conditions to those used with **DTE-PNP1** (Figure 4a). Similar experiments were conducted for the model PDI-containing *p*-nitrophenyl ester **PNP2** (Scheme S1). As shown in Table 1, replacing the acetate group of **DTE-PNP1** and **PNP1** by the carboxylated PDI unit in **DTE-PNP2** and **PNP2** slowed down the amidation process in most cases (CHCl_3_ and THF), in agreement with the well-known dependence of reaction rates with the steric hindrance around the acyl group.^30^ In spite of this, a clear positive effect derived from the introduction of the bulkier PDI group in **DTE-PNP2** and **PNP2**: the difference in reactivity between the open and closed isomers of **DTE-PNP2** was not only preserved, but it slightly increased relative to **DTE-PNP1**: *k*_DTE‑PNP2c_^obs^/*k*_DTE‑PNP2o_^obs^ = 7.5 and 14 in CHCl_3_ and THF, respectively, which further validates our strategy toward light-controlled amidation (Table 1, Figures 4b and S12). Consequently, these results denote that photoswitchable DTE-PNP active esters could be used to optically control chemical functionalization by light-modulated amide bond formation.

To illustrate this concept, we utilized **DTE-PNP2** to photoregulate the functionalization of an amino-containing polymer with PDI chromophores through amidation. High-molecular weight poly­(ethylenimine) (**PEI**, *M*_n_ ∼ 250,000 g mol^–1^) containing both reactive primary and secondary amines was chosen as a substrate in these experiments, which had to be dissolved in CHCl_3_ (*c* = 0.15 mM) because of its low solubility in THF. The resulting solution was then incubated with **DTE-PNP2**_**o**_ (*c* = 1.9 mM; 13 PDI units per polymer chain) for 15 min at two different illumination conditions: (1) in the dark, and (2) under UV irradiation (λ_exc_ = 365 nm, 10 mW cm^–2^). Subsequent treatment allowed us to isolate the reacted polymer, whose PDI content was determined by ^1^H NMR in each case (Figure 4c,d). It must be noted that only PDI and **PEI** resonances were observed in the ^1^H NMR spectra measured for the treated polymer samples, while no DTE signals were detected (Figure S13). Consequently, the PDI content determined by ^1^H NMR analysis should not come from **DTE-PNP2** molecules unspecifically physisorbed to the polymer, but to DTE-free PDI units covalently attached to **PEI** via amide bond formation. Clearly, minor covalent PDI labeling occurred in the dark due to the low aminolysis rate constant of the initial open state of **DTE-PNP2**, which therefore marginally reacted with **PEI** (1.6 PDI units attached per polymer chain, 12% labeling efficiency). In contrast, efficient PDI functionalization was accomplished upon UV irradiation by generating the more reactive closed isomer of **DTE-PNP2** in situ, which largely underwent aminolysis with **PEI** and yielded a strongly red-colored polymer sample (11.2 PDI units anchored per polymer chain, 89% labeling efficiency).

### 2.3 Light-Control of Polymer Network Formation via Photoswitchable Amidation

One of the major applications of dual-wavelength gated photoligation reactions is the light-induced control of polymer network formation, eventually aiming to develop advanced processes for polymer printing and manipulation.^13–16^ Thus, herein we investigated the use of our photoswitchable active esters to photoregulate the curing of amino-functionalized polymers by cross-linking with two colors of light. Thus, dimer **DTE-PNP3** was devised as a cross-linker agent, synthesized by simply tethering two units of phenol **DTE-NO**_**2**_ to tetradecandioyl chloride (see Supporting Information). A long alkyl chain was chosen as a spacer between the terminal DTE-PNP units of the dimer because of two principal motives: (1) to provide sufficient flexibility as to minimize steric and conformational constraints that could disfavor amidation reactivity for the two separate DTE-PNP moieties; and (2) to prevent detrimental effects on the photocyclization efficiency of the appended DTE groups. The latter has been reported for DTE aggregates linked through short tethers, where intramolecular energy transfer processes between nearby closed and open units lead to incomplete photoconversion.^31^ Thanks to the long C12 spacer introduced, this issue was not observed for **DTE-PNP3** in CHCl_3_, which preserved the high ring-closing conversion of monomer **DTE-PNP1**, producing 90% of closed DTE units under irradiation at 365 nm (Scheme S2, Table S3 and Figure S14). As a result, UV illumination of the initial open isomer **DTE-PNP3**_**o**_ efficiently led to the sequential photocyclization of its two DTE units to predominantly produce the fully closed form **DTE-PNP3**_**c**_ in the photostationary state. This behavior, in combination with the quantitative ring-opening efficacy measured for **DTE-PNP3**_**c**_ under red-light illumination, allowed repetitive photoswitching of **DTE-PNP3** in CHCl_3_ between its fully open and closed states with low degradation effects (Figure S14).

When subjected to aminolysis with model 1-dodecylamine at room temperature, **DTE-PNP3** also reproduced the reactivity behavior previously determined for monomer **DTE-PNP1** (Figure 5a). In this case, kinetic studies were conducted in CHCl_3_, as it was the solvent of choice for the subsequent polymer cross-linking experiments (see below). As shown in Table 1 and Figure 5b, clear modulation of amidation reactivity was observed between the open and closed isomers of **DTE-PNP3**, reaching a value of *k*_DTE‑PNP3c_^obs^/*k*_DTE‑PNP3o_^obs^ = 8.5. As already noted for PDI-containing **DTE-PNP2**, replacing the acetate moiety of **DTE-PNP1** by a bulkier acyl group in **DTE-PNP3** amplified the rate constant difference between the two states of the photoswitchable active ester, though at the expense of slowing down amide bond formation. A similar steric hindrance effect was found for the reactivity of reference active ester dimer **PNP3**, which was prepared by coupling of tetradecandioyl chloride with two equivalents of *p*-nitrophenol (see Supporting Information).

**Figure 5:**
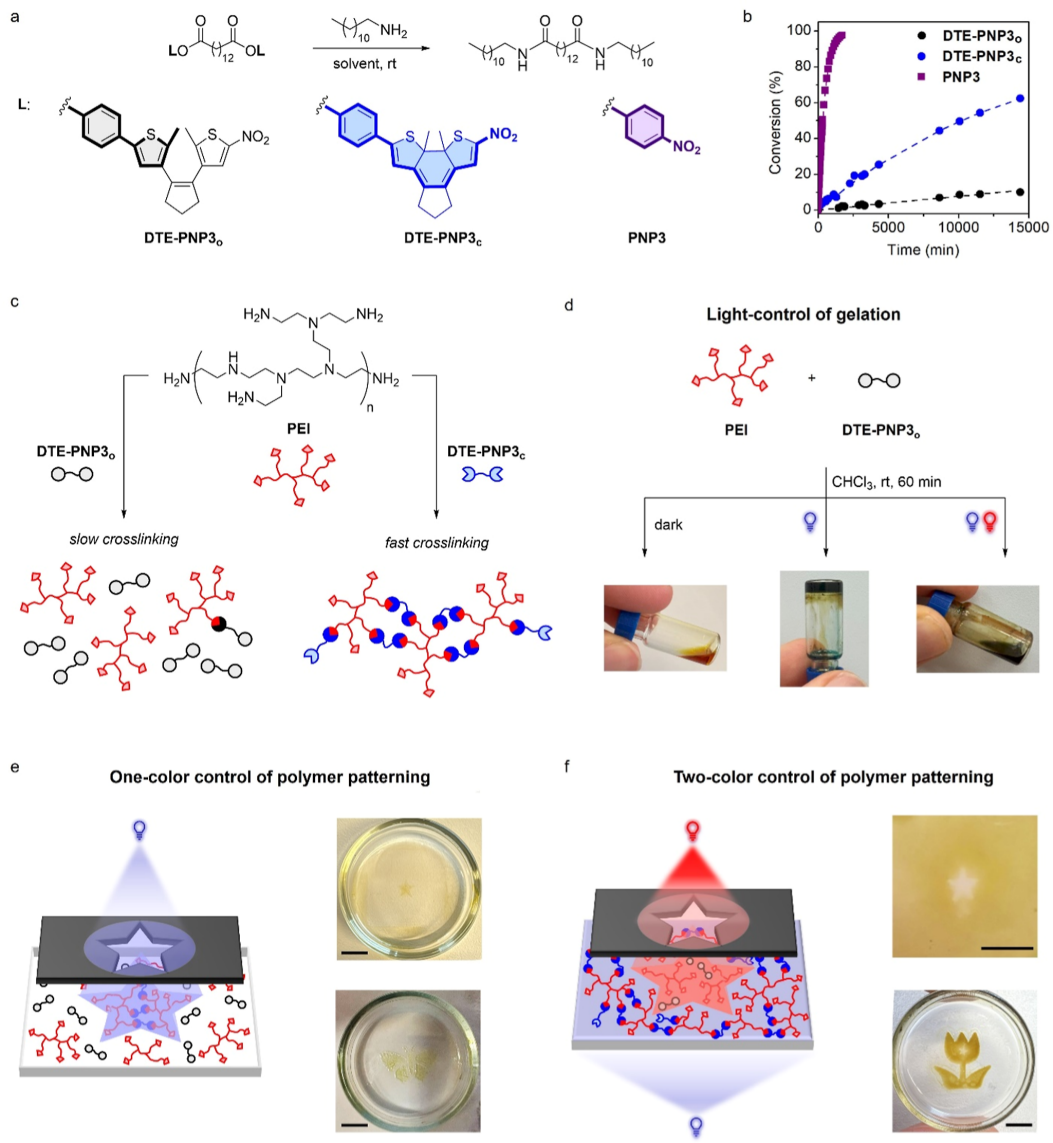
(a) Aminolysis reaction between **DTE-PNP3** (*c* = 2.5 × 10^–3^ M) or **PNP3** (*c* = 2.5 × 10^–3^ M) with 1-docecylamine (*c* = 0.10 M) at room temperature. (b) Evolution of aminolysis conversion with reaction time for **DTE-PNP3**_**o**_, **DTE-PNP3**_**c**_ and **PNP3** in CHCl_3_ at room temperature. Data points are experimental data obtained from ^1^H NMR measurements (see the Supporting Information for further details), and lines are fits to a pseudo-first order kinetic model (fit error <5%). Full reaction conversion for **DTE-PNP3**_**o**_ was not even observed after 30 days. (c) Modulation of **PEI** cross-linking by amidation reaction with the open and closed states of **DTE-PNP3**. (d) Light-color control of **PEI** gelation in CHCl_3_ at room temperature by reaction with **DTE-PNP3**_**o**_ under different illumination conditions: (left) in the dark; (middle) after UV irradiation (λ_max_ = 365 nm and 30 mW cm^–2^); and (right) after simultaneous UV (λ_max_ = 365 nm and 30 mW cm^–2^) and red-light irradiation (λ_max_ = 625 nm and 800 mW cm^–2^). (e) One-color control of solid polymer pattern formation by reaction of **PEI** with **DTE-PNP3**_**o**_ under UV illumination (λ_max_ = 365 nm and 3.8 mW cm^–2^) through a mask for 22 min (scale bar: 4.0 mm). (f) Two-color control of solid polymer pattern formation by reaction of **PEI** with **DTE-PNP3**_**o**_ under combined UV (λ_max_ = 365 nm and 3.8 mW cm^–2^) and red-light (λ_max_ = 650 nm and 500 mW cm^–2^) irradiation with differently shaped beams for 22 min (top) UV illumination of the whole field of view and red illumination through a star-shaped mask; and (bottom) UV and red illumination through flower- and star-shaped masks, respectively (scale bar: 4.0 mm). In (e,f) images of the polymer patterns created are shown after rinsing with CHCl_3_ to remove all unreacted species.

To exploit the difference in amidation reactivity exhibited by the two isomers of **DTE-PNP3** for the light-color control of polymer network formation, we investigated the cross-linking process of **PEI** as an amino-containing polymer (Figure 5c). In the first step, the photomodulation of **PEI** gelation in CHCl_3_ upon **DTE-PNP3**-induced covalent cross-linking was explored at room temperature. For this, gelation conditions were first optimized by amidation reaction with the reference cross-linker **PNP3**. In this way, we determined that chemical organogels could be rapidly produced in CHCl_3_ (*ca*. 5 min) by mixing appropriate amounts of **PEI** (*c* = 0.3 mM) and **PNP3** (*c* = 35 mM) (Figure S15). Then, analogous gelation experiments were conducted by replacing **PNP3** with **DTE-PNP3**_**o**_ under three different illumination conditions: (1) in the dark; (2) under UV irradiation (λ_exc_ = 365 nm, 30 mW cm^–2^); and (3) under combined UV (λ_exc_ = 365 nm, 30 mW cm^–2^) and red-light irradiation (λ_exc_ = 625 nm, 800 mW cm^–2^). As shown in Figure 5d, **PEI** gelation was selectively promoted under sole UV irradiation in *ca*. 60 min; i.e., by photoconverting **DTE-PNP3**_**o**_ to the more reactive isomer **DTE-PNP3**_**c**_. The time required for cross-linker photocyclization (*ca*. 30 min at the experimental conditions used) should largely account for the longer gelation time measured in this case relative to the **PNP3**-induced process, together with the slower amidation kinetics of **DTE-PNP3**_**c**_. On the other hand, no gelation was observed in the dark when starting from the less reactive isomer **DTE-PNP3**_**o**_ (even after 72 h), or under simultaneous UV and red-light illumination with high visible-to-UV intensity ratio to warrant predominant formation of **DTE-PNP3**_**o**_ in the photostationary state (at least for 6 h). These results were corroborated by monitoring the gelation kinetics of separate **PEI**:**DTE-PNP3**_**o**_ and **PEI**:**DTE-PNP3**_**c**_ mixtures with rheometric measurements, where an estimate of the gel time can be obtained from the crossover of the storage (G′) and loss (G″) moduli (Figure S16). Whereas no gel point was determined for the **PEI**:**DTE-PNP3**_**o**_ sample after 50 min, G′/G″ crossover was observed for the **PEI**:**DTE-PNP3**_**c**_ mixture just after 8 min, thereby indicating the fast gelation capacity of the closed state of the cross-linker. Overall, our gelation experiments demonstrate that toggling between the two different states of photoswitchable DTE-PNP esters allows the two-color control of solid polymer network formation, which is triggered with UV irradiation and inhibited by illumination with red light.

In the next stage, we applied the light-color controlled cross-linking reaction between **PEI** and **DTE-PNP3** to fabricate polymer patterns. After preliminary optimization of the experimental conditions, we established that, when depositing a liquid layer (*ca*. 1 mm in height) of a CHCl_3_ solution of **PEI** (*c* = 0.75 mM) and **DTE-PNP3**_**o**_ (*c* = 10 mM) onto glass, irradiation with UV light (λ_exc_ = 365 nm, 3.8 mW cm^–2^) at room temperature for *ca.* 20 min promoted the formation of an insoluble, yellow-colored thin polymer layer by **DTE-PNP3**_**c**_ photogeneration and subsequent cross-linking (*ca*. 5 μm thick according to scanning electron microscopy). In contrast, no solid polymer network formation was observed in the dark even at longer incubation times (at least for 30 min) or when applying simultaneous two-color irradiation with UV (λ_exc_ = 365 nm, 3.8 mW cm^–2^) and red light (λ_exc_ = 625 nm and 500 mW cm^–2^) (Figures S17 and S18). Due to the high polymer content and, therefore, viscosity of the liquid formulations used, molecular diffusion was largely prevented during these experiments, which we exploited to create defined polymer patterns by conducting UV illumination through differently shaped masks; i.e., under one-color activation of **PEI** curing, which yielded spatially patterned polymer films exhibiting a characteristic yellow color (Figures 5e and S19). More interestingly, we also attempted polymer photopatterning under two-color irradiation (Figure S19). For this, we first conducted experiments where we induced generalized solid polymer film formation by irradiating the entire liquid formulation with UV light (λ_exc_ = 365 nm, 3.8 mW cm^–2^), while locally stopping amidation-promoted cross-linking by confined red illumination through a mask (λ_exc_ = 650 nm, 500 mW cm^–2^). As shown in Figure 5f, spatially patterned polymer layers were obtained in this way where the lack of yellow-colored polymer material was observed in the areas irradiated with red light, thus demonstrating the two-color antagonistic control of **PEI** cross-linking. In a further step, we took advantage of this behavior to produce other polymer patterns where both the UV and red illumination beams were patterned through masks, which illustrate the potential of the photoswitchable active esters developed in this work to modulate amidation reactions with high spatial precision (Figure 5f).

## 3 Conclusion

We introduce photoswitchable active esters for the light-color control of amide bond formation reactions with amines. Our approach relies on introducing a dithienylethene photoswitch within the structure of *p*-nitrophenyl active esters, whose open-closed photoisomerization allows reversibly turning on and off the activating effect of the *p*-nitro substituent on amidation reactivity. After reaction, the photoswitchable fragment of our active esters is released as a leaving group, while its acyl moiety remains attached to the reactive amine yielding a stable amide with no light-sensitivity. This behavior was successfully validated for three different types of photoswitchable phenyl esters: a model acetate ester, a functional ester bearing a transferable perylene diimide chromophore, and an ester dimer that was used as a cross-linker for amino-containing polymers. By enabling electronic communication between the phenyl ester and the *p*-nitro group through the conjugated backbone of the photocyclized dithienylethene unit, the closed isomer of these compounds showed 4- to 24-fold larger aminolysis rates than their open state when reacted with a model primary amine at room temperature. Taking advantage of this feature, two-color control of amide bond formation was demonstrated, as it could be promoted upon UV-induced photocyclization of the photoswitchable esters and dramatically slowed by back-photoisomerization to their initial open isomer with red light. In addition, although their amidation reactivity decreases relative to common active phenyl esters due to steric and electronic effects, our photoswitchable esters in their activated closed state undergo amide bond formation on the time scale of minutes to hours at ambient conditions. As a result, these compounds can be exploited to photocontrol relevant examples of amidation-based ligation processes, such as dye labeling, polymer gelation and polymer thin film patterning. Overall, these results illustrate the potential of photoswitchable active esters to accomplish the functionalization and manipulation of materials with enhanced spatiotemporal precision.

## Supplementary Material



## Notes

Additional information, figures and tables about the synthesis and characterization of photoswitchable active esters (PDF)

## Abbreviations

PNP: *p*-nitrophenyl
DTE: dithienylethene
PDI: perylene diimide
PEI: poly­(ethylenimine).
