# Supplementary material for: Photoswitchable Active Esters for the Control of Amide Bond Formation with Light
Source: JACS Au. 2025 Oct 3;5(10):5017–26. doi: 10.1021/jacsau.5c00930 (PMC12569660; doi:10.1021/jacsau.5c00930)
Supplement: Supplementary file 1 [file au5c00930_si_001.pdf]

Supporting Information

Photoswitchable Active Esters for the Control of Amide Bond Formation with Light

Marc Villabona,[a] Arnau Marco,[a] Rosa María Sebastián,[a] Christopher Barner-Kowollik,[b,c,*] Gonzalo Guirado[a,*] and Jordi Hernando[a,*]

[a] Departament de Química, Universitat Autònoma de Barcelona (UAB), Edifici C/n, Campus UAB, Cerdanyola del Vallès 08193, Spain

E-mail: gonzalo.guirado@uab.cat, jordi.hernando@uab.cat

[b] Institute of Nanotechnology (INT), Karlsruhe Institute of Technology (KIT), Hermann-von-Helmholtz-Platz 1, 76344 Eggenstein-Leopoldshafen, Germany

E-mail: christopher.barner-kowollik@kit.edu

[c] School of Chemistry and Physics, Centre for Materials Science, Queensland University of Technology (QUT), 2 George Street, Brisbane, QLD 4000, Australia

E-mail: christopher.barnerkowollik@qut.edu.au

Table of Contents

1. Materials and methods 3

2. Synthesis of DTE-PNP and PNP compounds 7

3. Photochemical characterization of DTE-PNP1 13

4. Kinetic studies of amidation of DTE-PNP1 and PNP1 16

5. Optical characterization of DTE-PNP2 19

6. Amidation studies with DTE-PNP2 and PNP2 21

7. Photochemical characterization of DTE-PNP3 22

8. Amidation studies with DTE-PNP3 and PNP3 25

9. NMR and IR spectra of selected compounds 28

10. References 38

1. Materials and methods

1.1. Materials

Acetyl chloride (Sigma-Aldrich), triethylamine (Sigma-Aldrich), 4-nitrophenol (Sigma-Aldrich), 12-aminododecanoic acid (Sigma-Aldrich), imidazole (Sigma-Aldrich), dicyclohexylcarbodiimide (Sigma-Aldrich), 4-dimethylaminopyridine (Sigma-Aldrich), tetradecandioyl dichloride (Sigma Aldrich), 1-dodecylamine (Sigma-Aldrich), sodium hydroxide (Fisher), hydrochloric acid (Fisher), anhydrous sodium sulfate (Fisher), tetrahydrofuran (Fisher), dichloromethane (Fisher), chloroform (Fisher), hexane (Scharlab), ethyl acetate (Scharlab), diethyl ether (Fisher), toluene (Fisher), acetonitrile (Fisher), dimethylsulfoxide (Fisher), ethanol (Fisher), chloroform-*d* (Euroisotop), tetrahydrofuran-d_8 (Euroisotop), dimethylsulfoxide-d_6 (Euroisotop), acetonitrile-d_3 (Euroisotop) and toluene-d_8 (Euroisotop) were purchased and used as received unless specified. Tetrahydrofuran and dichloromethane were dried with molecular sieve 4 Å, -8+12 (ca. 2 mm) beads (Sigma-Aldrich). Thin layer chromatography (TLC) was performed using silica gel 60 precoated aluminum plates (0.20 mm thickness). Flash column chromatography was performed using silica gel (230-400 mesh).

1.2. Experimental methods

NMR spectroscopy: NMR spectra were recorded on Bruker DPX250 (250 MHz for ^1H), BRUKER 300 MHz AscendTM (300 MHz for ^1H), DPX360 (360 MHz for ^1H), AvanceIII 400NB (400 MHz for ^1H) and Bruker System 600 Ascend LH (600 MHz for ^1H) spectrometers. The δ-scale was normalized relative to the residual solvent signal for ^1H NMR and ^{13}C NMR ($CDCl_3$ (7.26 ppm for ^1H; 77.2 ppm for ^{13}C), CD_3CN (1.94 ppm for ^1H; 118.3 and 1.3 ppm for ^{13}C), DMSO-d_6 (2.50 ppm for ^1H; 39.5 ppm for ^{13}C), CD_2Cl_2 (5.32 ppm for ^1H; 54.0 ppm for ^{13}C), THF-d_8 (3.58 and 1.73 ppm for ^1H; 67.6 and 25.4 ppm for ^{13}C) and toluene-d_8 (7.09, 7.01, 6.97 and 2.08 ppm for ^1H; 137.5, 128.9, 128.0, 125.1 and 20.4 ppm for ^{13}C)). The abbreviations used to describe signal multiplicities are: s (singlet), d (doublet), t (triplet), q (quadruplet), p (pentuplet), h (sextet) and m (multiplet).

IR spectroscopy: IR-ATR spectra were recorded in a Bruker Tensor 27 Golden Gate spectrometer with a diamond tip.

Mass spectrometry: Mass spectra were recorded in a Bruker Esquire 3000+ spectrometer using ESI and timsTOF Pro 2 spectrometer using ESI.

UV-vis absorption spectroscopy: UV-vis absorption spectra were recorded on an Agilent HP 8453 spectrophotometer. Samples were measured in Hellma Analytics

quartz high precision cells with a path length of 10 mm or 0.33 mm at ambient temperature.

Fluorescence spectroscopy: Fluorescence spectra were recorded by means of a custom-made spectrofluorometer using a cw laser at λ_{exc} = 532 nm (Z-laser) as an excitation source. Emitted photons were detected using an Andor ICCD camera coupled to a spectrograph. All the emission spectra registered were corrected by the wavelength dependence of the spectral response of the detection system. In all cases, spectroscopic quality solvents and 1 cm quartz cuvettes were used. Temperature was controlled using a refrigerated circulator bath (Huber MPC-K6) connected to the sample holder. Fluorescence quantum yields were determined using the standard method[1] for highly diluted solutions of the compounds of interest to prevent self-absorption processes (absorption < 0.05 at the excitation wavelength) and relative to *N*,*N'*-bis(butyl)-1,6,7,12-tetra-(4-*tert*-butylphenoxy)perylene-3,4:9,10-tetracarboxylicdiimide in CH_2Cl_2 (Φ_{fl} = 1).[2] The same excitation wavelength was used for both the sample of interest and the standard in all these measurements. When monitoring the fluorescence of **DTE-PNP2$_c$**, very low irradiation intensities were used to minimize direct dithienylethene ring-opening.

Scanning electron microscopy (SEM): SEM images of polymer films were registered with a FEI Quanta 650 ESEM microscope applying a 5.0 kV voltage. All the samples were sputtered with a layer of about 5 nm of Pt prior to SEM imaging.

Photoisomerization experiments: DTE-PNP photoisomerization was investigated by NMR and UV-vis absorption spectroscopies using different types of irradiation sources: a Nd:YAG pulsed laser (Brilliant, Quantel, λ_{exc}= 355) or a 3W LED with $\lambda_{exc,max}$ = 365 nm (LED365) for photocyclization; a diode cw laser at λ_{exc} = 650 nm (SciTech) or a 100 W LED with $\lambda_{exc,max}$ = 625 nm for photocycloreversion (LED625). Figure S1 shows the emission spectra of LED365 and LED625, which were determined in an Andor ICCD camera coupled to a spectrograph. The irradiation power of all these sources was measured using a Gentec TPM-300 power meter and, in the case of LED365 and LED625, it was tuned by varying the applied current and/or the separation distance with respect to the illuminated sample. The composition of the photostationary state (PSS) obtained upon irradiation of DTE-PNP compounds with UV light (LED365) was determined by ^{1}H NMR in the deuterated solvents of interest (PSS_{o-c}). From this data and the UV-vis absorption spectra of the open isomer and PSS_{o-c} in the same solvent, the UV-vis absorption spectrum of the closed isomer was estimated using equation S1. In this equation $Abs_{DTE\text{-}PNP_o}(\lambda)$, $Abs_{DTE\text{-}PNP_c}(\lambda)$ and $Abs_{PSS_{o\text{-}c}}(\lambda)$ are the absorption of the open, closed and photostationary states at a given wavelength, respectively, and χ_o and χ_c are the molar fractions of the open and closed isomers in PSS_{o-c} determined by ^{1}H NMR:

$$\mathrm{Abs}_{\mathrm{DTE\text{-}PNP_c}}(\lambda)=\frac{\mathrm{Abs}_{\mathrm{PSS_{o\text{-}c}}}(\lambda)-\chi_o\,\mathrm{Abs}_{\mathrm{DTE\text{-}PNP_o}}(\lambda)}{\chi_c} \quad (1)$$

Photoisomerization quantum yields were determined by monitoring the variation of the UV-vis absorption spectra of DTE-PNP compounds in the solvent of interest upon irradiation with UV (for photocyclization, at λ_{exc} = 355 nm) or visible light (for photocycloreversion, at λ_{exc} = 650 nm) and applying a previously reported kinetic model to the absorption data.[3] In the case of **DTE-PNP3** that undergoes two consecutive photocyclization/photocycloreversion steps to interconvert between its open and closed states, we assumed each of these two steps to be independent and take place with the same quantum yield, as previously observed for other diarylethene dimers with long, flexible alkyl linkers.[4] The irradiation intensities used in our photoisomerization quantum yield experiments were determined by monitoring the photocyclization and photocycloreversion processes of 1,2-bis(2-methyl-5-phenyl-3-thienyl)perfluorocyclopentene in hexane as a reference ($\Phi_{o\text{-}c}$ = 0.59 and $\Phi_{c\text{-}o}$ = 0.013).[5]

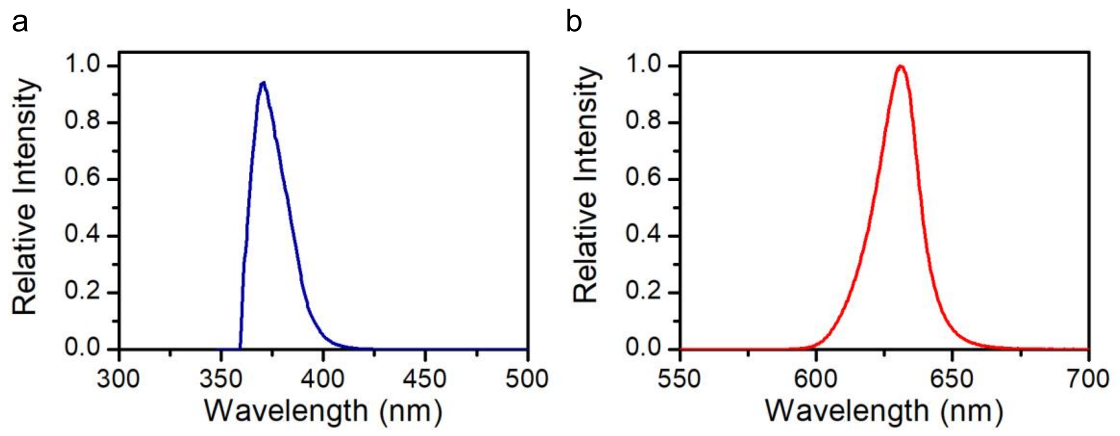

Figure S1. Emission spectra of (a) LED365 and (b) LED625.

Kinetic measurements: The kinetics of the aminolysis reaction between PNP, DTE-PNP_o and DTE-PNP_c compounds with 12-dodecylamine was investigated at room temperature and ambient conditions in aprotic solvents ($CHCl_3$, THF and acetonitrile). The initial concentrations used were: c_{amine} = 0.10 M and c_{ester} = 5.0 10^{-3} M, except for the experiments with dimers **DTE-PNP3$_o$** and **DTE-PNP3$_c$**, where $c_{\mathbf{DTE\text{-}PNP3}}$ = 2.5 10^{-3} M to make the initial amount of ester groups be 5.0 10^{-3} M. For measurements with DTE-PNP_c compounds, the closed-state molecules of the photoswitchable esters were prepared by irradiation at λ_{max} = 365 nm until reaching the corresponding photostationary states (DTE-PNP_c content > 80%, see Table S1). For **PNP1** in THF and acetonitrile and **PNP2** in THF, reaction kinetics was monitored by taking UV-vis absorption spectra of the reaction mixture every 10 s using a cuvette with a path length of 0.33 mm. Analysis was performed at an absorption wavelength

where absorbance remained below 1.0 during the whole experiment despite the high PNP concentration used (e.g., at λ_{abs} = 430 nm for **PNP1** in Figure S5). For the rest of the cases that underwent slower aminolysis reactions, kinetics was monitored by ^{1}H NMR spectroscopy and amidation conversion was obtained by comparing the integrals in the low-field region of the initial ester and of the resulting leaving group as well as of the protons at the α-position to the nitrogen atom of the amide product (e.g., see Figure S4 for **DTE-PNP1$_{o}$**). In all cases, apparent pseudo-first order rate constants (k^{obs}) were obtained by fitting the spectral data to a monoexponential function. The error of the k^{obs} values obtained from the fits was below 5% in all cases.

Rheometry measurements: Gelation kinetics was investigated using a MCR 102e Anton Paar rheometer, an aluminum plate to deposit the sample, and initial gap of 0.5 mm between the sample and the instrument.

2. Synthesis of DTE-PNP and PNP compounds

2.1. Synthesis of 4-nitrophenyl acetate (PNP1)

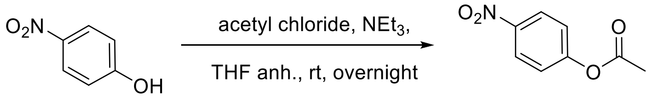

PNP1

0.610 g of 4-nitrophenol (4.38 mmol) were dissolved in 20 ml of anhydrous THF. Then, 0.35 mL of acetyl chloride (4.90 mmol) and 0.80 mL of triethylamine (5.74 mmol) were added, and the mixture was stirred overnight at room temperature. Once the reaction was finished, 20 mL of diethyl ether were added and the organic phase was washed twice with 20 mL of a 1 M NaOH solution. The organic phase was dried with anhydrous Na_2SO_4, the resulting solid was filtered off, and the solvent was removed under vacuum to obtain 0.761 g of pure **PNP1** (4.20 mmol, 95% yield). The ^{1}H NMR spectrum of this compound matched previously reported data.[6]

^{1}H NMR (600 MHz, $CDCl_3$) δ 8.28 (d, *J* = 9.1 Hz, 2H), 7.29 (d, *J* = 9.1 Hz, 2H), 2.35 (s, 3H).

2.2. Synthesis of 1-(2-methyl-5-(4-acetoxy)phenyl)thien-3-yl)-2-(2-methyl-5-nitrothien-3-yl)cyclopentene (DTE-PNP1$_o$)

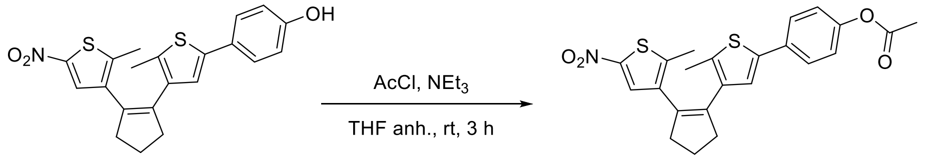

DTE-NO$_2$ **DTE-PNP1$_o$**

0.101 g of **DTE-NO$_2$**[7] (0.25 mmol) were added onto a solution containing 0.10 mL of triethylamine (0.71 mmol) and 0.10 mL of acetyl chloride (1.40 mmol) in 5 mL of THF. The mixture was stirred for 3 hours, after which 10 mL of water and 10 mL of diethyl ether were poured. The organic phase was washed with water once and then dried over anhydrous Na_2SO_4 and filtered. After solvent was removed under vacuum, the crude was purified via flash column chromatography (silica gel, hexanes/EtOAc, 9:1) to obtain 0.099 g of **DTE-PNP1$_o$** (0.23 mmol, 89% yield).

^{1}H NMR (400 MHz, $CDCl_3$): δ (ppm) 7.69 (s, 1H), 7.47 (d, *J* = 8.6 Hz, 2H), 7.07 (d, *J* = 8.7 Hz, 2H), 6.90 (s, 1H), 2.84 (t, *J* = 7.4 Hz, 2H), 2.78 (t, *J* = 7.5 Hz, 2H), 2.31 (s, 3H), 2.10 (p, *J* = 7.5 Hz, 2H), 2.00 (s, 3H), 1.98 (s, 3H).

^{13}C NMR (101 MHz, $CDCl_3$): δ (ppm) 169.6, 150.0, 147.8, 144.3, 139.8, 137.7, 136.5, 135.9, 134.8, 132.5, 132.1, 129.8, 126.5, 123.7, 122.1, 38.7, 38.5, 23.0, 21.2, 15.3, 14.6.

IR (ATR, cm^{-1}): 2916, 2844, 2051, 1750, 1499, 1422, 1367, 1321, 1191, 1165, 1093, 1015, 949, 909, 835, 817, 800, 753, 736, 664.

HRMS (ESI): m/z calcd for $C_{23}H_{21}NNaO_4S_2^+$: 462.0804 [M-Na]$^+$; found: 462.0803.

2.3. Synthesis of PNP2

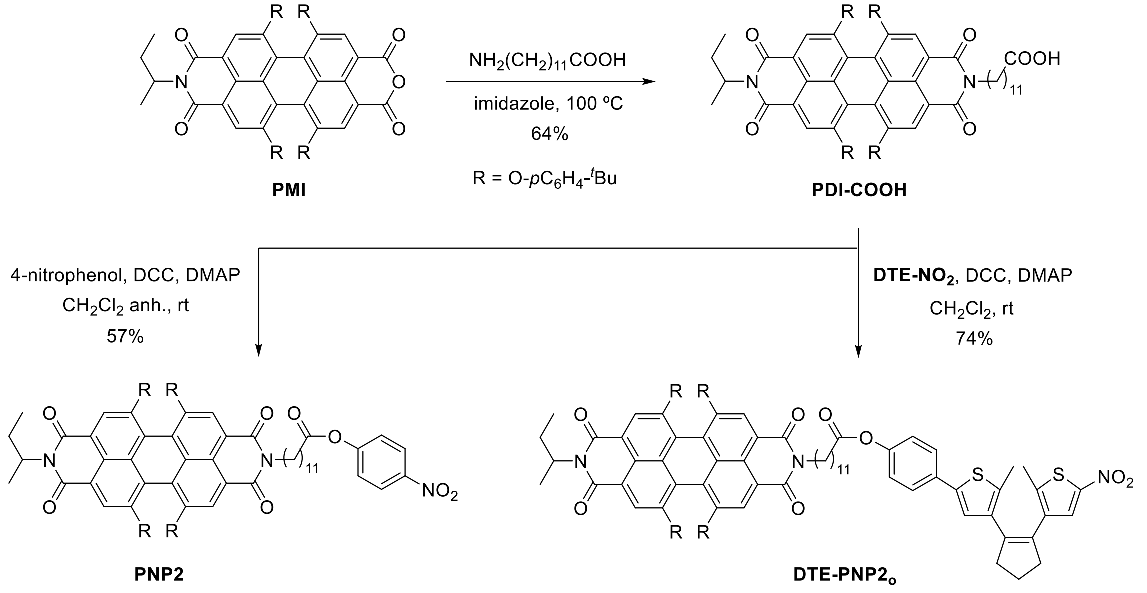

Scheme S1. Synthesis of **PNP2** and **DTE-PNP2$_o$** from perylene monoanhydride **PMI**, which was prepared as previously reported in our group.[2]

Synthesis of N-(sec-butyl)-N-(11-carboxyundecyl)-1,6,7,12-tetra(4-tert-butyl phenoxy)perylene-3,4:9,10-tetracarboxylic-3,4-anhydride-9,10-imide (PDI-COOH)

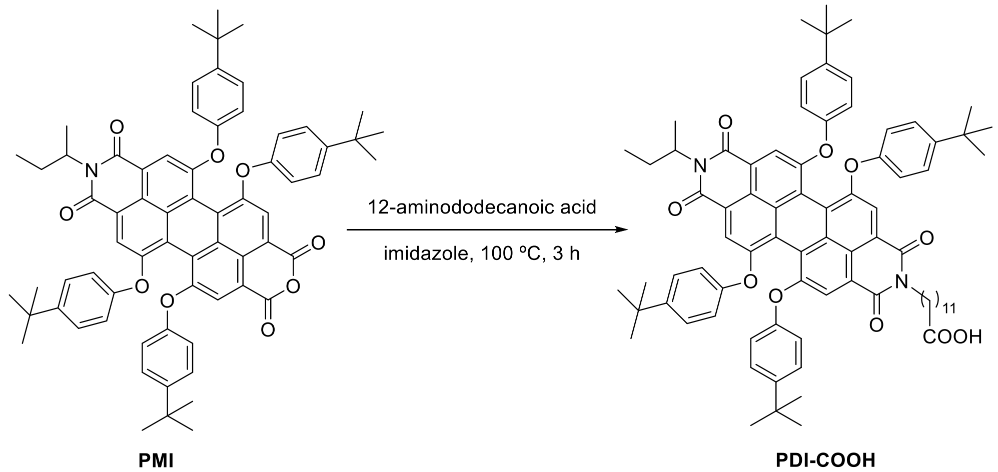

0.183 g of anhydride **PMI**[2] (0.18 mmol), 0.081 g of 12-aminododecanoic acid (0.38 mmol) and 2.002 g of imidazole were heated at 100 ºC for 3 hours in a 50 mL round bottom flask under argon atmosphere. Afterwards, the reaction mixture was cooled down to room temperature and it was dissolved in the minimum amount of ethanol required. Then, 100 mL of an aqueous 2 M HCl solution were added, and the mixture was stirred for 30 min. The precipitate was filtered under vacuum and dried in a vacuum oven at 130 ºC for 3 hours. The crude was purified via flash column chromatography (silica gel, $CHCl_3$) to obtain 0.140 g of **PDI-COOH** (0.11 mmol, 64% yield).

^{1}H NMR (400 MHz, $CDCl_3$): δ (ppm) 8.22 (s, 2H), 8.20 (s, 2H), 7.23 (d, *J* = 8.5 Hz, 8H), 6.82 (d, *J* = 8.5 Hz, 8H), 5.12 (h, *J* = 6.4 Hz, 1H), 4.09 (t, *J* = 7.6 Hz, 2H), 2.32 (t, *J* = 7.5 Hz, 2H), 2.14 (m, 1H), 1.86 (m, 1H), 1.73 – 1.53 (m, 4H), 1.50 (d, *J* = 6.8 Hz, 3H), 1.38 – 1.19 (m, 50H), 0.86 (t, *J* = 7.4 Hz, 3H).

^{13}C NMR (101 MHz, $CDCl_3$): δ (ppm) 164.1, 163.6, 156.2, 156.0, 153.0, 153.0, 152.9, 147.4, 133.1, 132.9, 126.9, 126.8, 123.0, 122.5, 120.7, 120.4, 120.0, 119.9, 119.7, 119.6, 119.5, 119.4, 119.4, 51.5, 40.8, 34.5, 33.8, 31.6, 29.8, 29.8, 29.6, 29.5, 29.4.

IR (ATR, cm^{-1}): 2928, 2325, 2050, 1696, 1658, 1586, 1503, 1461, 1410, 1339, 1281, 1210, 1172, 1110, 1014, 877, 832, 803, 750, 702, 668.

HRMS (ESI): m/z calcd for $C_{80}H_{87}N_2O_{10}^-$: 1235.6366 $[M]^-$; found: 1235.6369.

*Synthesis of N-(sec-butyl)-N-(12-(4-nitrophenoxy)-12-oxododecyl)-1,6,7,12-tetra(4-tert-butylphenoxy)perylene-3,4:9,10-tetracarboxylic-3,4-anhydride-9,10-imide (**PNP2**)*

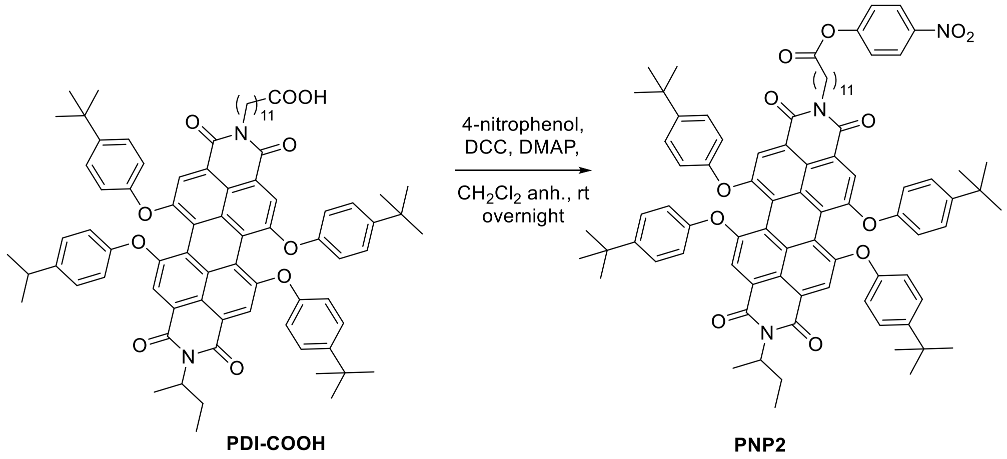

0.039 g of perylene diimide **PDI-COOH** (0.03 mmol), 0.015 g of 4-nitrophenol (0.11 mmol), 0.017 g of DCC (0.82 mmol) and 0.005 g of DMAP (0.04 mmol) were stirred overnight in 10 mL of anhydrous CH_2Cl_2. After this time, the solvent was removed

under vacuum and the crude purified via flash column chromatography (silica gel, hexanes/EtOAc, 9:1) to obtain 0.025 g of **PNP2** (0.02 mmol, 57% yield).

^{1}H NMR (400 MHz, $CDCl_3$): δ (ppm) 8.26 (d, *J* = 9.1 Hz, 2H), 8.22 (s, 2H), 8.20 (s, 2H), 7.26 (d, *J* = 9.1 Hz, 2H), 7.24 – 7.20 (m, 8H), 6.85 – 6.80 (m, 8H), 5.12 (m, 1H), 4.09 (t, *J* = 7.6 Hz, 2H), 2.57 (t, *J* = 7.5 Hz, 2H), 2.14 (m, 1H), 1.86 (m, 1H), 1.78 – 1.62 (m, 4H), 1.50 (d, *J* = 6.9 Hz, 3H), 1.44 – 1.14 (m, 50H), 0.86 (t, *J* = 7.4 Hz, 3H).

^{13}C NMR (101 MHz, $CDCl_3$): δ (ppm) 171.4, 164.1, 163.6, 156.2, 156.0, 155.7, 153.1, 153.0, 147.4, 145.4, 133.1, 132.9, 126.8, 126.8, 125.3, 123.1, 122.6, 122.5, 120.8, 120.4, 120.1, 119.9, 119.7, 119.6, 119.5, 119.5, 51.5, 40.8, 34.5, 34.5, 31.6, 29.8, 29.6, 29.6, 29.5, 29.4, 29.3, 29.2, 28.2, 27.2, 26.7, 24.9, 18.3, 11.6.

IR (ATR, cm^{-1}): 2930, 2857, 2051, 1765, 1695, 1656, 1587, 1524, 1503, 1461, 1433, 1410, 1341, 1283, 1209, 1173, 1109, 1014, 878, 832, 804, 749, 702, 667.

HRMS (ESI): m/z calcd for $C_{86}H_{92}N_3O_{12}^{+}$: 1358.6676 $[M-H]^{+}$; found: 1358.6661

2.4. Synthesis of *N*-(*sec*-butyl)-*N*-(12-(4-(5-methyl-4-(2-(2-methyl-5-nitrothio phen-3-yl)cyclopent-1-en-1-yl)thiophen-2-yl)phenoxy)-12-oxododecyl)-1,6,7,12-tetra(4-*tert*-butylphenoxy)perylene-3,4:9,10-tetracarboxylic-3,4-anhydride-9,10-imide (DTE-PNP2$_o$)

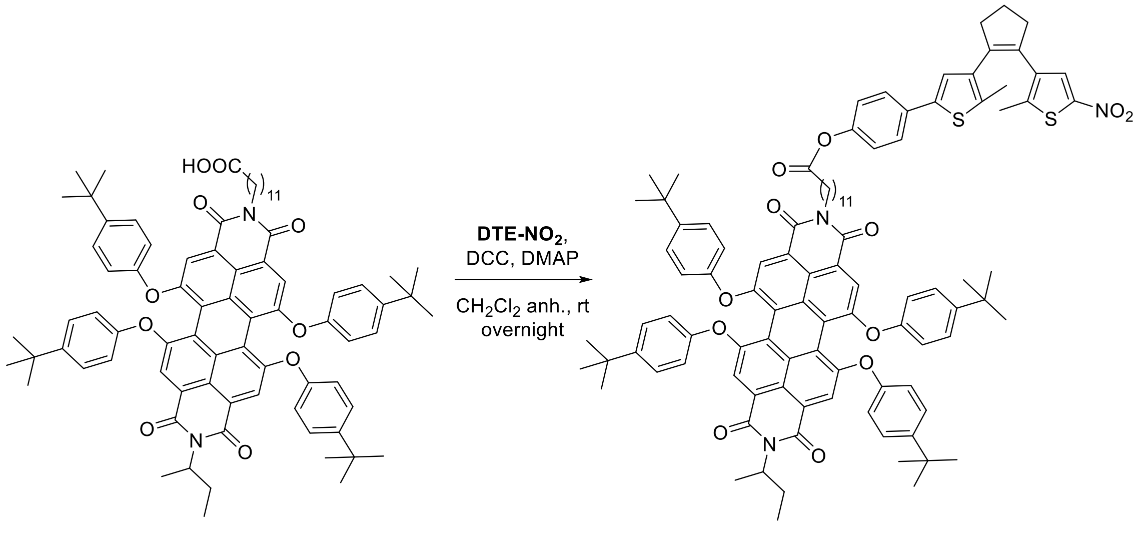

PDI-COOH **DTE-PNP2$_o$**

0.233 g of **PDI-COOH** (0.19 mmol), 0.101 g of **DTE-NO$_2$** (0.25 mmol), 0.152 g of DCC (3.9 mmol) and 0.005 g of DMAP (0.04 mmol) were stirred overnight in 10 mL of anhydrous CH_2Cl_2. Afterwards, the solvent was removed under vacuum, and the crude was purified via flash column chromatography (silica gel, hexanes/EtOAc 4:1) to obtain 0.231 g of **DTE-PNP2$_o$** (0.14 mmol, 74% yield).

^{1}H NMR (360 MHz, $CDCl_3$): δ (ppm) 8.22 (s, 2H), 8.20 (s, 2H), 7.69 (s, 1H), 7.45 (d, *J* = 8.7 Hz, 2H), 7.23 (d, *J* = 8.4 Hz, 8H), 7.04 (d, *J* = 8.8 Hz, 2H), 6.90 (s, 1H), 6.83

(d, J = 8.6 Hz, 8H), 5.12 (h, J = 6.3 Hz, 1H), 4.10 (t, J = 7.6 Hz, 2H), 2.89 – 2.74 (m, 4H), 2.53 (t, J = 7.5 Hz, 2H), 2.11 (m, 1H), 1.99 (s, 3H), 1.97 (s, 3H), 1.86 (m, 1H), 1.78 – 1.61 (m, 4H), 1.50 (d, J = 6.9 Hz, 3H), 1.43 – 1.18 (m, 50H), 0.86 (t, J = 7.3 Hz, 3H).

^{13}C NMR (91 MHz, $CDCl_3$): δ (ppm) 172.4, 164.1, 163.6, 156.1, 156.0, 153.0, 153.0, 150.1, 147.8, 147.4, 144.3, 139.9, 137.7, 136.5, 135.9, 134.7, 133.1, 132.9, 132.5, 132.0, 129.8, 126.8, 126.8, 126.5, 123.6, 123.0, 122.5, 122.2, 120.7, 120.4, 120.0, 119.9, 119.7, 119.6, 119.5, 119.4, 119.4, 51.5, 40.8, 38.7, 38.5, 34.5, 31.8, 31.6, 29.8, 29.8, 29.6, 29.6, 29.5, 29.4, 29.4, 29.2, 28.2, 27.3, 26.6, 25.1, 23.0, 18.3, 15.3, 14.6, 11.6.

IR (ATR, cm^{-1}): 2927, 2857, 2361, 2325, 2051, 1981, 1754, 1694, 1656, 1586, 1503, 1461, 1410, 1335, 1284, 1209, 1168, 1109, 1014, 879, 832, 803, 750, 703, 668.

HRMS (ESI): m/z calcd for $C_{101}H_{106}N_3O_{12}S_2^+$: 1616.7212 [M-H]$^+$; found: 1616.7214.

2.5. Synthesis of bis(4-nitrophenyl) tetradecanedioate (PNP3)

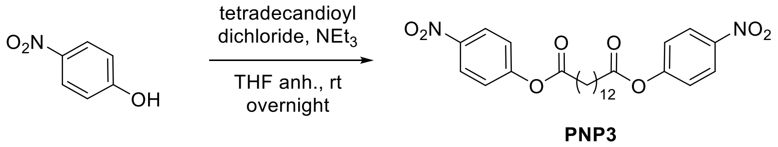

0.523 g of 4-nitrophenol (3.76 mmol) were dissolved in 20 ml of anhydrous THF. Then, 0.469 g of tetradecandioyl chloride (1.59 mmol) and 0.60 mL of triethylamine (4.30 mmol) were added, and the mixture was stirred overnight at room temperature. Once the reaction was finished, 20 mL of diethyl ether were added and the organic phase was washed twice with 20 mL of a 1 M NaOH solution. The organic phase was dried with anhydrous Na_2SO_4 and the solid was filtered. After the solvent was removed under vacuum, the crude was purified by flash column chromatography (silica gel, hexanes/EtOAc, 9:1) to obtain 0.641 g of **PNP3** (1.31 mmol, 80% yield).

^{1}H NMR (400 MHz, $CDCl_3$) δ 8.27 (d, J = 9.1 Hz, 4H), 7.27 (d, J = 9.1 Hz, 4H), 2.60 (t, J = 7.4 Hz, 4H), 1.76 (p, J = 7.4 Hz, 4H), 1.47 – 1.23 (m, 16H).

^{13}C NMR (101 MHz, $CDCl_3$) δ 171.4, 155.7, 145.4, 125.3, 122.6, 34.5, 29.7, 29.5, 29.4, 29.2, 24.9.

IR (ATR, cm^{-1}): 3119, 3089, 2918, 2850, 1752, 1620, 1592, 1531, 1488, 1472, 1417, 1379, 1344, 1308, 1252, 1204, 1193, 1128, 1104, 1011, 923, 866, 854, 757, 717, 679, 645, 575, 528, 496, 450.

HRMS (ESI): m/z calcd for $C_{26}H_{32}N_2NaO_8^+$: 523.2051 [M-Na]$^+$; found: 523.2055.

2.5. Synthesis of bis(4-(5-methyl-4-(2-(2-methyl-5-nitrothiophen-3-yl) cyclopent-1-en-1-yl)thiophen-2-yl)phenyl) tetradecanedioate (DTE-PNP3o)

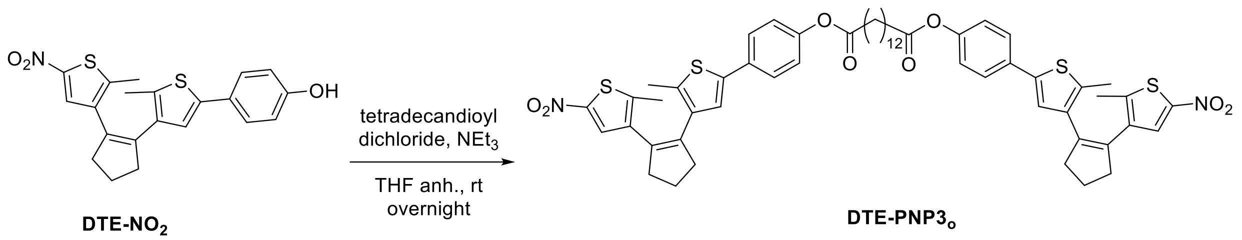

0.190 g of **DTE-NO2** (0.47 mmol) were dissolved in 10 ml of anhydrous DCM. Then, 0.062 g of tetradecandioyl chloride (0.21 mmol) and 0.10 mL of triethylamine (0.72 mmol) were added, and the mixture was stirred overnight at room temperature. Once the reaction was finished, the organic phase was washed twice with 10 mL of a 1 M HCl solution. The organic phase was dried with anhydrous Na_2SO_4 and the solid was filtered. After the solvent was removed under vacuum, the crude was purified by flash column chromatography (silica gel, hexanes/EtOAc, 9:1) to obtain 0.201 g of **DTE-PNP3o** (0.20 mmol, 95% yield).

^{1}H NMR (600 MHz, $CDCl_3$) δ 7.69 (s, 2H), 7.46 (d, *J* = 8.7 Hz, 4H), 7.06 (d, *J* = 8.7 Hz, 4H), 6.90 (s, 2H), 2.84 (td, *J* = 7.5, 2.3 Hz, 4H), 2.79 (td, *J* = 7.3, 2.2 Hz, 4H), 2.56 (t, *J* = 7.5 Hz, 4H), 2.10 (p, *J* = 7.8 Hz, 4H), 2.00 (s, 6H), 1.98 (s, 6H), 1.75 (p, *J* = 7.5 Hz, 4H), 1.41 (p, *J* = 7.2 Hz, 4H), 1.37 – 1.27 (m, 12H).

^{13}C NMR (151 MHz, CDCl3) δ 172.5, 150.1, 147.8, 144.3, 139.9, 137.7, 136.5, 136.0, 134.8, 132.5, 132.0, 129.8, 126.5, 123.7, 122.2, 38.7, 38.5, 34.5, 29.7, 29.6, 29.4, 29.2, 25.1, 23.0, 15.3, 14.6.

IR (ATR, cm^{-1}): 2922, 2850, 1754, 1500, 1421, 1321, 1201, 1165, 1092, 1016, 916, 832, 816, 753, 736, 660, 500, 464.

HRMS (ESI): m/z calcd for $C_{56}H_{60}N_2NaO_8S_4^+$: 1039.3125 [M-Na]$^+$; found: 1039.3120.

3. Photochemical characterization of DTE-PNP1

Table S1. Photochemical properties of **DTE-PNP1** in different solvents.[a]

Solvent	$\lambda_{abs,max,o}$ (ε)[b]	$\lambda_{abs,max,c}$ (ε)[b]	$PSS_{o\text{-}c}$[c]	$PSS_{c\text{-}o}$[d]	$\Phi_{o\text{-}c}$[e]	$\Phi_{c\text{-}o}$[f]
**Toluene**	297 (23.5)	628 (13.1)	0.97	1.00	0.75	0.0077
**$CHCl_3$**	300 (23.7)	648 (13.2)	0.95	1.00	0.16	0.0069
**THF**	298 (25.4)	632 (12.6)	0.96	1.00	0.53	0.0089
**ACN**	299 (23.5)	638 (13.2)	0.82	1.00	0.055	0.0057
**DMSO**	303 (23.2)	657 (9.3)	0.72	1.00	0.037	0.0054

[a] Photochemical properties were investigated at 365 nm (for photocyclization) and 625/650 nm (for photocycloreversion) based on the UV-vis absorption spectra of the open and closed isomers of **DTE-PNP1**. However, these wavelengths could be further refined by action plot measuring.[8,9] [b] Absorption maximum in nm of the open ($\lambda_{abs,max,o}$) and closed ($\lambda_{abs,max,c}$) isomers. For the closed isomer, the spectral maximum is given for its visible absorption band. The extinction coefficients in 10^3 M^{-1} cm^{-1} are indicated in parenthesis. [c] Molar fraction of the closed isomer after prolonged irradiation at 365 nm. The values were determined by ^1H NMR measurements. [d] Molar fraction of the open isomer after irradiation at 625 nm determined by UV-vis absorption measurements. [e] Ring-closing quantum yields upon irradiation at 355 nm. [e] Ring-opening quantum yields upon irradiation at 650 nm.

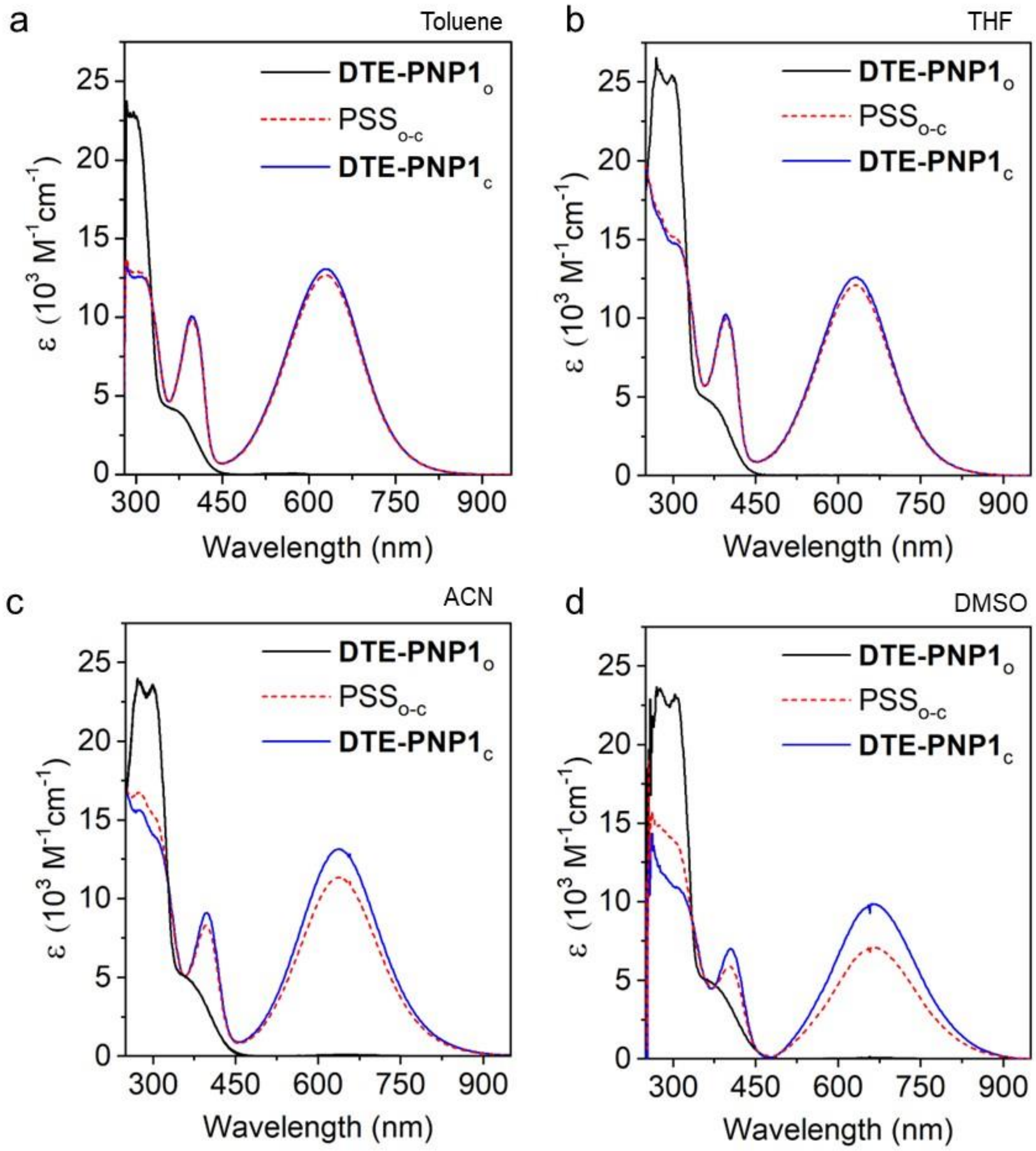

Figure S2. DTE-PNP1 extinction coefficients in (a) toluene, (b) THF, (c) acetonitrile and (d) DMSO for the open isomer **DTE-PNP1$_o$** (black), the closed isomer **DTE-PNP1$_c$** (blue), and the PSS reached under photoexcitation at 365 nm (dashed red).

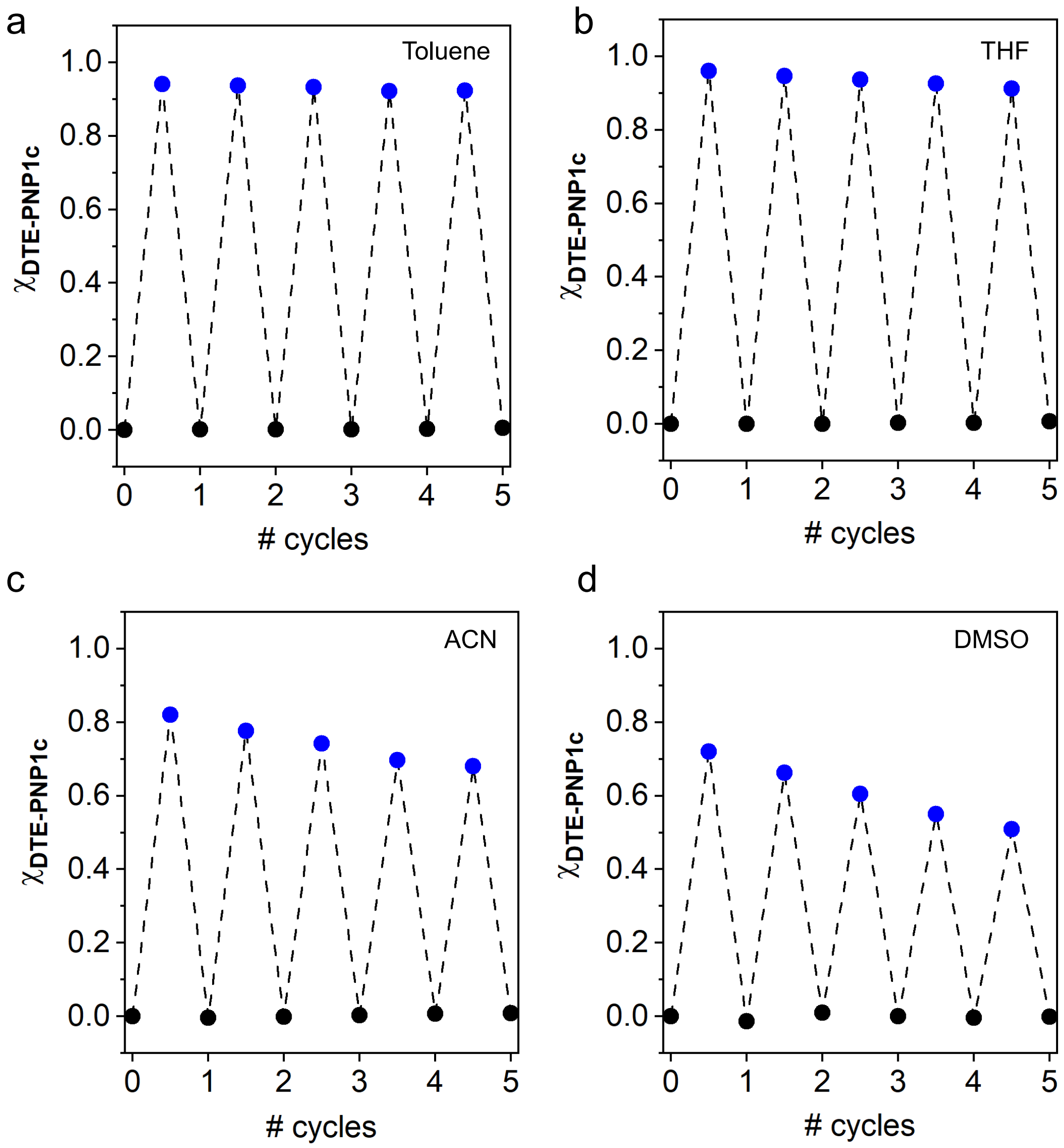

Figure S3. Variation of the molar fraction of the closed isomer of **DTE-PNP1** when it was subjected to 5 cycles of consecutive irradiation at 365 nm and 625 nm in (a) toluene, (b) THF, (c) acetonitrile and (d) DMSO.

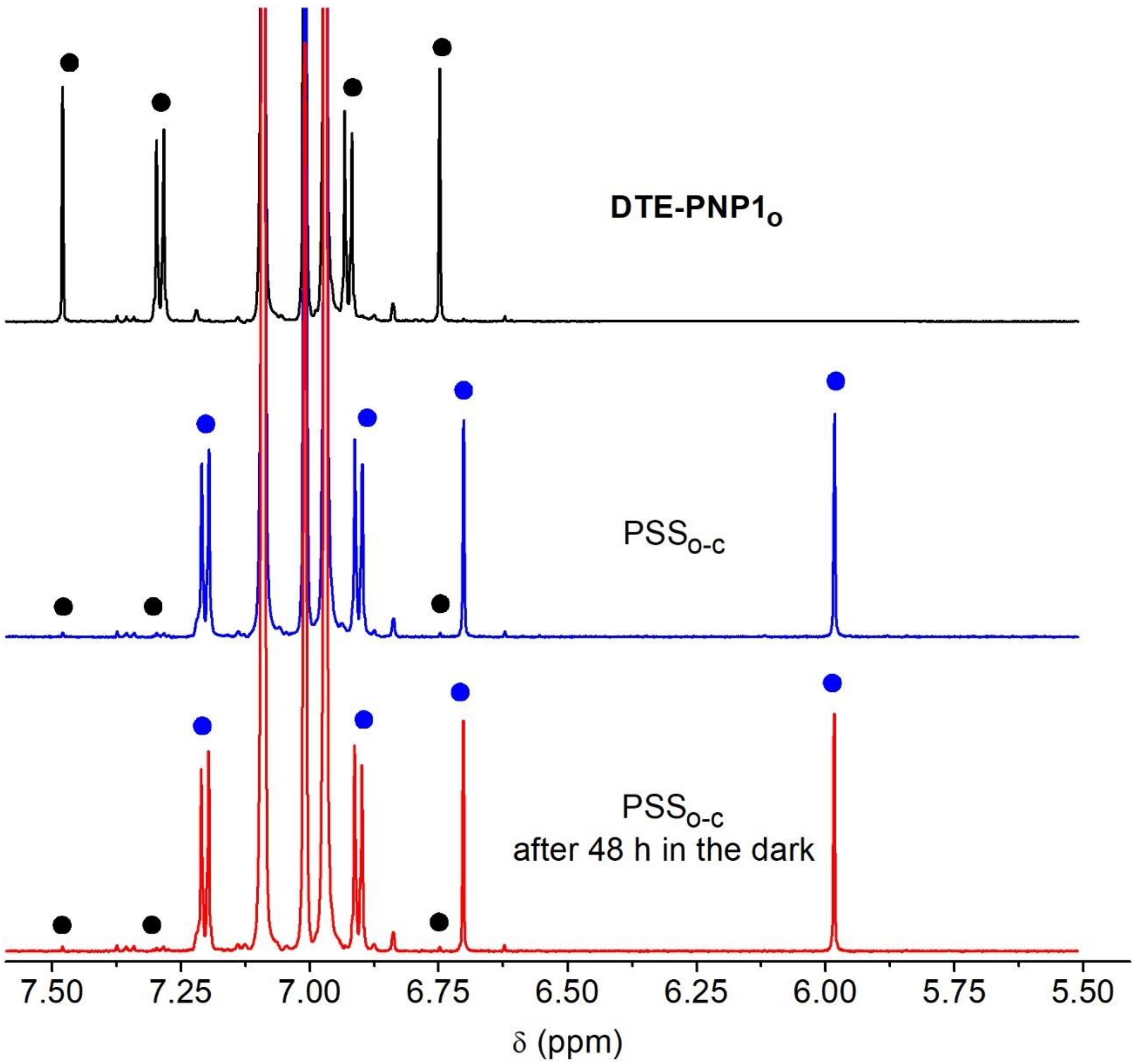

Figure S4. Downfield region of the ^{1}H NMR spectra (600 MHz, toluene-d_8) of **DTE-PNP1$_o$** and of the photostationary state produced at λ_{exc} = 365 nm, which contains 97% of the closed isomer. For the photostationary state, data is shown just after irradiation ($PSS_{o\text{-}c}$) and after storage in the dark for 48 h at room temperature, where no effects of thermal back-isomerization were observed.

4. Kinetic studies of amidation of DTE-PNP1 and PNP1

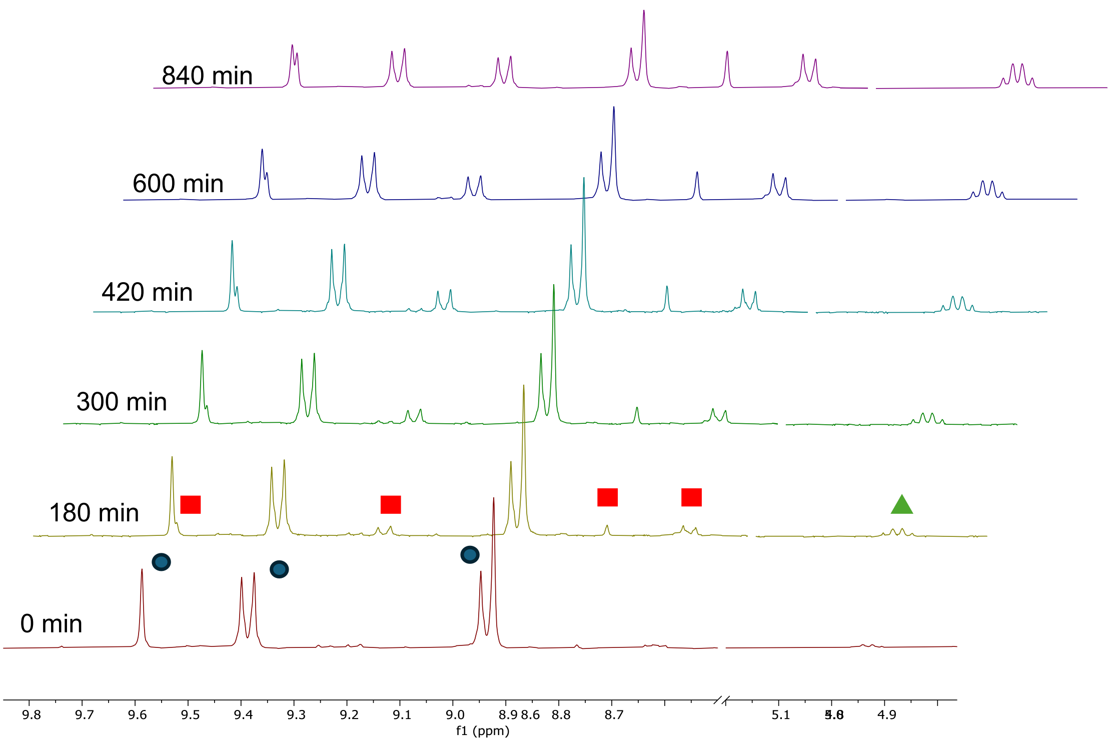

Figure S5. Variation of the ^{1}H NMR (250 MHz, CD_3CN, 298K) spectrum of the reaction mixture of **DTE-PNP1₀** (5 mM) and 1-dodecylamine (100 mM). Black dots indicate the signals corresponding to the initial **DTE-PNP1₀** ester, red squares correspond to the signals of the **DTE-NO₂** leaving group of the amidation reaction, and the green triangle labels the signal arising from the protons at the α-position to the nitrogen atom of the amide product.

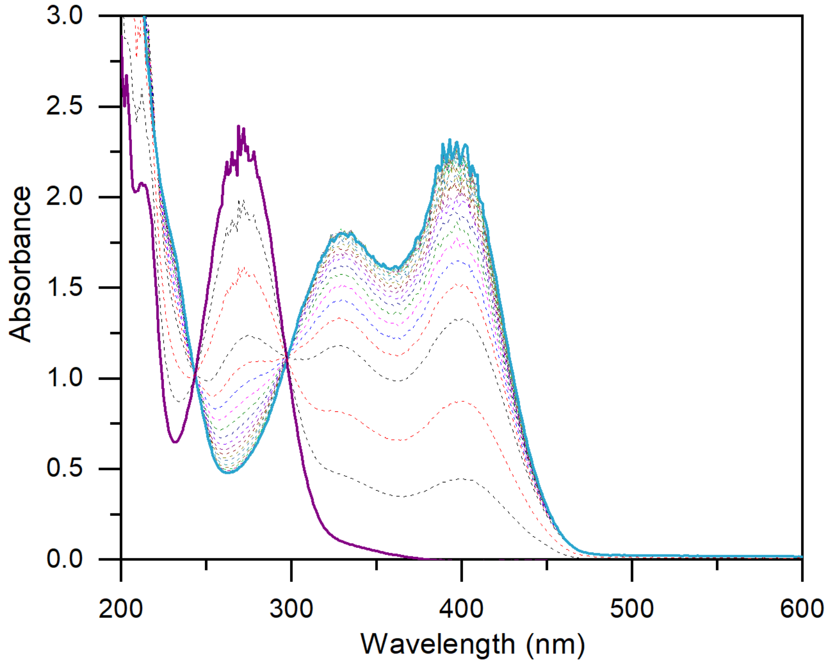

Figure S6. Variation of the UV-vis absorption spectrum of the reaction mixture of **PNP1** (5 mM) and 1-dodecylamine (100 mM) in acetonitrile. Spectra were measured every 10 s using a cuvette with a path length of 0.33 mm. Analysis was performed at λ_{abs} = 430 nm, where absorbance remained below 1.0 during the whole experiment despite the high **PNP1** concentration used.

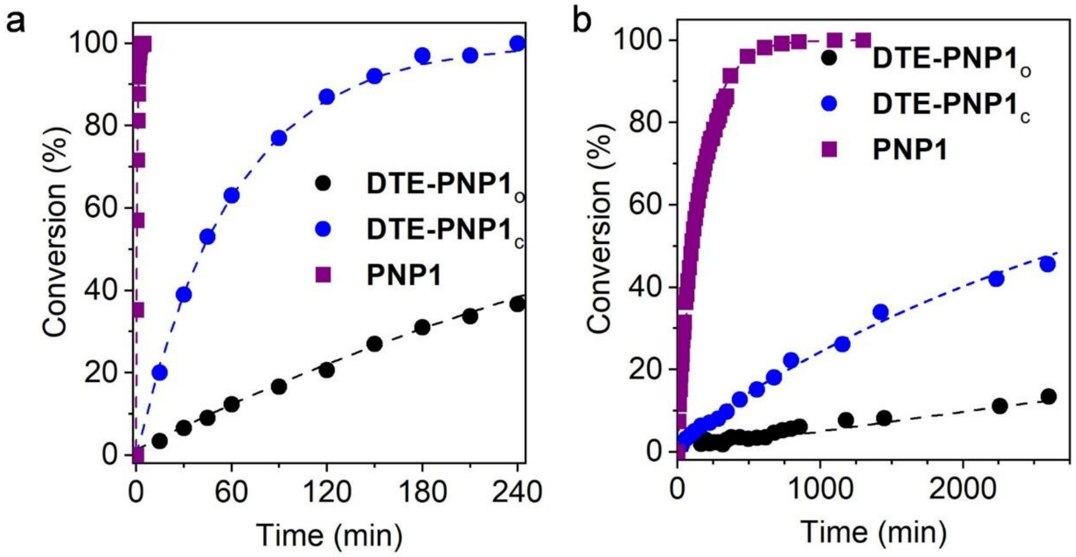

Figure S7. Time evolution of the conversion of the aminolysis reaction of **DTE-PNP1$_o$**, **DTE-PNP1$_c$** or **PNP1** (5 mM) with 1-dodecylamine (100 mM) in (a) THF and (b) chloroform at room temperature. Data points are experimental data obtained from ^{1}H NMR or UV-vis absorption spectra, and lines are fits to a pseudo-first order kinetic model (fit error < 5%). Full conversion was observed for **DTE-PNP1$_o$** in THF after 2 days, and for **DTE-PNP1$_c$** in chloroform after 10 days.

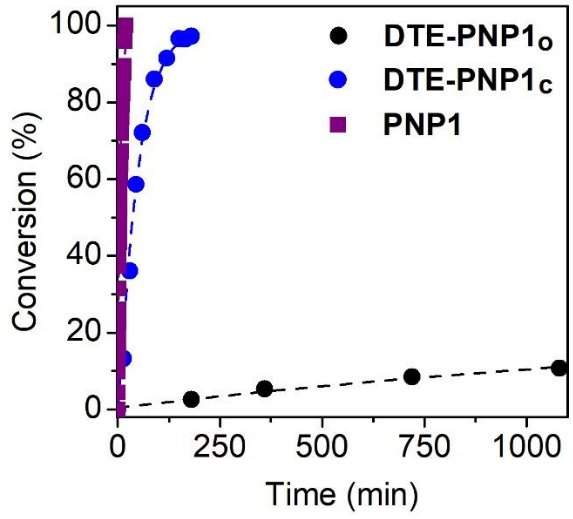

Figure S8. Time evolution of the conversion of the aminolysis reaction of **DTE-PNP1o**, **DTE-PNP1c** or **PNP1** (5 mM) with dihexylamine (100 mM) in acetonitrile at room temperature. Data points are experimental data obtained from ^{1}H NMR or UV-vis absorption spectra, and lines are fits to a pseudo-first order kinetic model (fit error < 5%). The apparent pseudo-first order rate constants obtained from these fits are: $k^{obs}_{\mathbf{DTE\text{-}PNP1o}} = 8.4\cdot10^{-6}$ s^{-1}, $k^{obs}_{\mathbf{DTE\text{-}PNP1c}} = 3.3\cdot10^{-4}$ s^{-1}, and $k^{obs}_{\mathbf{PNP1}} = 2.3\cdot10^{-3}$ s^{-1}.

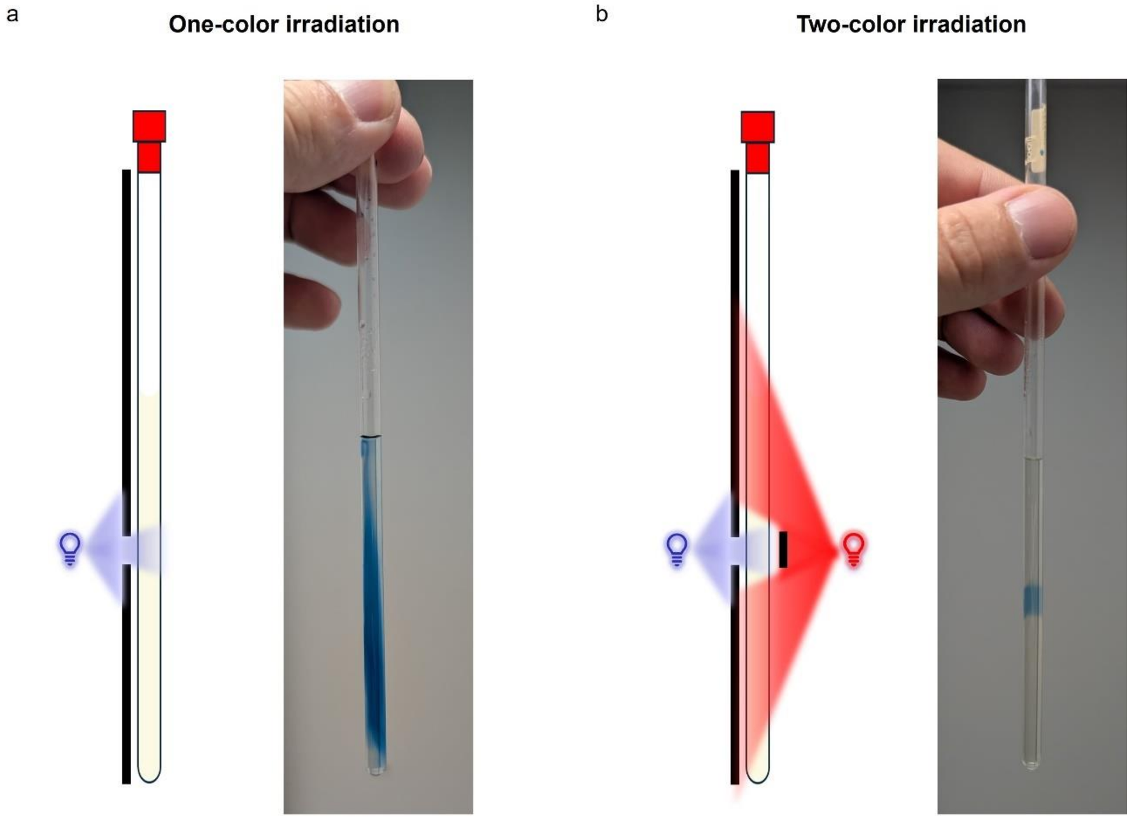

Figure S9. Patterned irradiation of a **DTE-PNP1**$_o$ solution in CD_3CN within an NMR tube using one or two excitation wavelengths: (a) irradiation at λ_{max} = 365 nm (0.017 mW cm^{-2}) for 5 min through a *ca.* 0.5 cm lateral aperture; and (b) combined photoexcitation for 5 min at λ_{max} = 365 nm (0.017 mW cm^{-2}) through a *ca.* 0.5 cm lateral aperture, and at λ_{max} = 625 nm (400 mW cm^{-2}), with which the whole NMR tube was fully illuminated from the opposite lateral side except for a *ca.* 0.5 cm segment that was at the same height as the 365 nm illumination aperture. Because of molecular diffusion, the blue-colored **DTE-PNP1**$_c$ molecules generated upon UV irradiation did not remain confined within the illuminated volume element in (a), but they migrated to other areas of the solution. In contrast, complementary irradiation with red light of the rest of the sample in (b) induced back-photoisomerization to the colorless **DTE-PNP1**$_o$ state, thereby enabling localization of the **DTE-PNP1**$_c$ molecules within the 365 nm illuminated region. All experiments were conducted with no stirring or shaking.

5. Optical characterization of DTE-PNP2

Table S2. Optical properties of **DTE-PNP2** in THF and $CHCl_3$.[a]

Isomer	$\lambda_{abs,max,o}$ (ε)[b]	$\lambda_{abs,max,c}$ (ε)[b]	$PSS_{o\text{-}c}$[c]	$PSS_{c\text{-}o}$[d]	$\Phi_{o\text{-}c}$[e]	$\Phi_{c\text{-}o}$[f]	$\Phi_{f,o}$[g]	$\Phi_{f,c}$[g]
**$CHCl_3$**	286 (72.4), 585 (47.4)	585 (54.9) 649 (7.8)	0.78	1.00	0.043	0.0089	0.89	0.60
**THF**	284 (77.5), 570 (43.6)	570 (51.2), 632 (12.0)	0.89	1.00	0.066	0.013	0.80	0.19

[a] Photochemical properties were investigated at 365 nm (for photocyclization) and 625/650 nm (for photocycloreversion) based on the UV-vis absorption spectra of the open and closed isomers of **DTE-PNP2**. However, these wavelengths could be further refined by action plot measuring.[8,9] [b] Absorption maximum in nm of the open ($\lambda_{abs,max,o}$) and closed ($\lambda_{abs,max,c}$) isomers. Two different values are given in each case corresponding to the absorption bands of the DTE (open isomer ~ 285 nm; closed isomer ~ 640 nm) and PDI (~575 nm) units of **DTE-PNP2**. The extinction coefficients in 10^3 M^{-1} cm^{-1} are indicated in parenthesis. [c] Molar fraction of the closed isomer in the photostationary state achieved under continuous irradiation at 365 nm, which was measured by ^{1}H NMR. [d] Molar fraction of the open isomer after irradiation at 625 nm determined by UV-vis absorption measurements. [e] Ring-closing quantum yield measured upon irradiation at 355 nm. This value is affected by the competitive absorption of the PDI and DTE units of **DTE-PNP2$_o$** at the excitation wavelength (ε_{PDI} ~ ε_{DTEo} at 355 nm), leading to an apparent decrement in $\Phi_{o\text{-}c}$. [f] Ring-opening quantum yield measured under 650 nm irradiation. [g] Fluorescence quantum yield of the open ($\Phi_{f,o}$) and closed ($\Phi_{f,o}$) isomers (λ_{exc} = 532 nm). For both isomers, the same emission spectrum at $\lambda_{f,max}$ = 617 nm ($CHCl_3$) and 602 nm (THF) was measured.

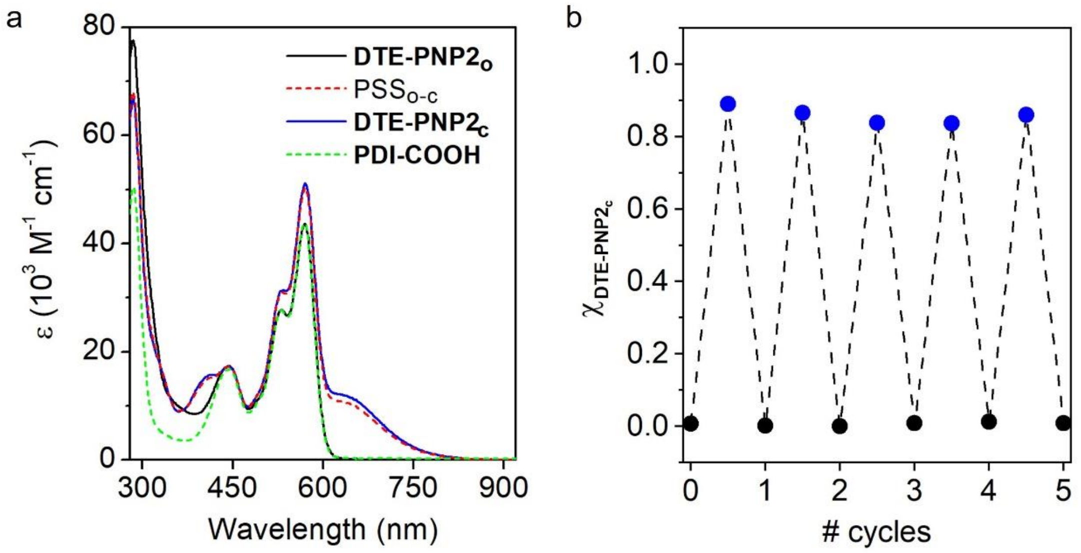

Figure S10. a) UV-vis absorption spectra of the open and closed isomers of **DTE-PNP2** in THF, as well as of the photostationary state mixture obtained upon irradiation of **DTE-PNP1$_o$** at 365 nm ($PSS_{o\text{-}c}$). For comparison purposes, the absorption spectrum of the DTE-free model emitter **PDI-COOH** in THF is also shown. b) Variation of the molar fraction of the closed isomer of **DTE-PNP2** when it was subjected to 5 cycles of consecutive irradiation at 365 nm and 625 nm in THF.

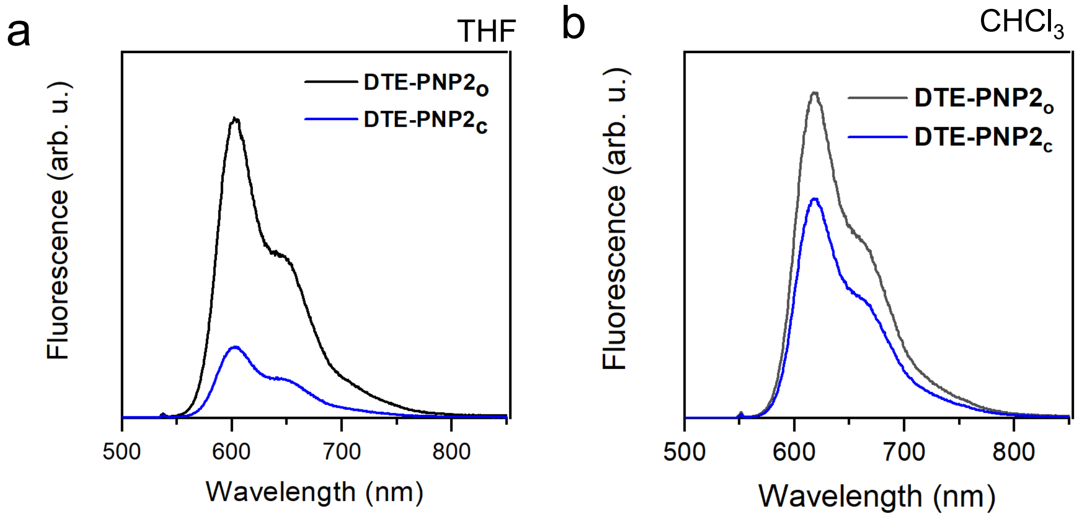

Figure S11. Fluorescence spectra of **DTE-PNP2$_o$** and **DTE-PNP2$_c$** in (a) THF and (b) $CHCl_3$ (c = 2.0 μM, λ_{exc} = 532 nm).

6. Amidation studies with DTE-PNP2 and PNP2

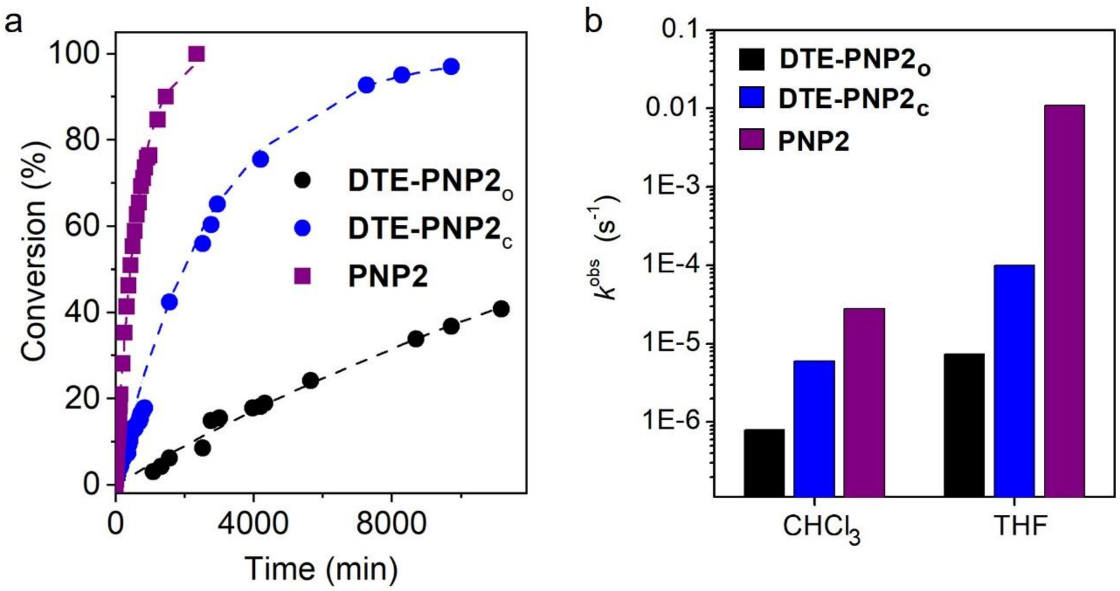

Figure S12. (a) Time evolution of the conversion of the aminolysis reaction of **DTE-PNP2$_o$**, **DTE-PNP2$_c$** or **PNP2** (5 mM) with 1-dodecylamine (100 mM) in chloroform at room temperature. Data points are experimental data obtained from ^{1}H NMR spectra and lines are fits to a pseudo-first order kinetic model (fit error < 5%). (b) Pseudo-first order rate constants for the aminolysis reaction of **DTE-PNP2$_o$**, **DTE-PNP2$_c$** and **PNP2** in chloroform and THF at room temperature.

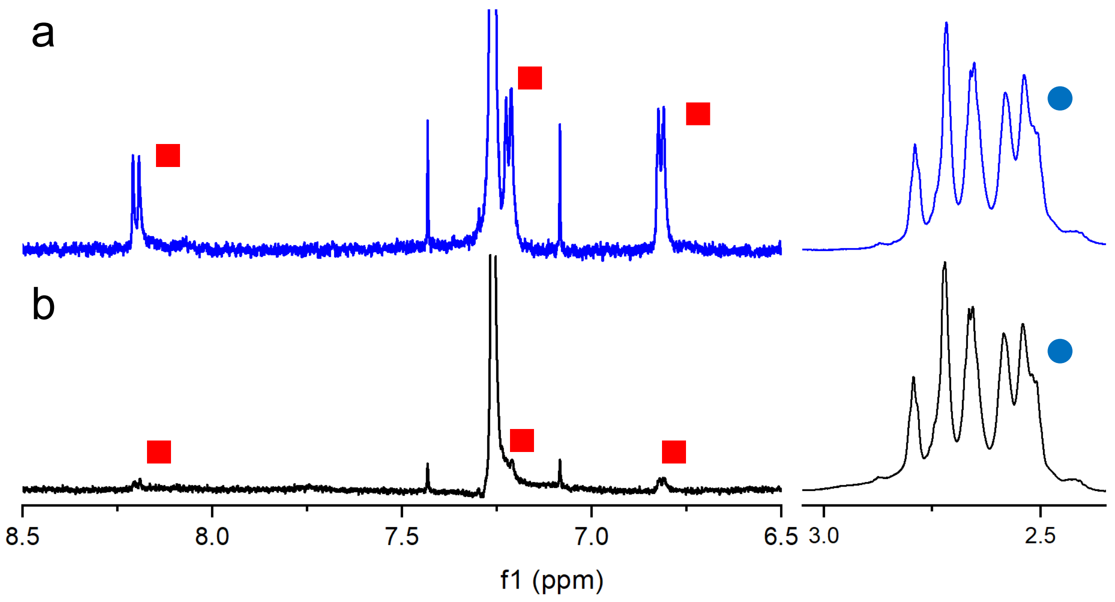

Figure S13. ^{1}H NMR spectra (600 MHz, $CDCl_3$) of the PDI-functionalized PEI polymers obtained by amidation reaction between **PEI** (*c* = 0.15 mM) and **DTE-PNP2$_o$** (*c* = 1.9 mM) at room temperature and two different irradiation conditions: (a) in the dark for 15 min, and (b) under irradiation at 365 nm and 10 mW cm^{-2} for 15 min. Red squares are used to indicate the resonances arising from the PDI-anchored units, and blue circles are used to label the signals from PEI backbone. For clarity, the intensity of the low-field region (6.5 – 8.5 ppm) where the aromatic PDI resonances fall was enlarged 20x. It must be noted that no DTE signals were observed in either of the two ^{1}H NMR spectra, which indicates that the PDI resonances detected should not come from **DTE-PNP2** molecules unspecifically physisorbed to the polymer, but to DTE-free PDI units covalently attached to **PEI** via amide bond formation.

7. Photochemical characterization of DTE-PNP3

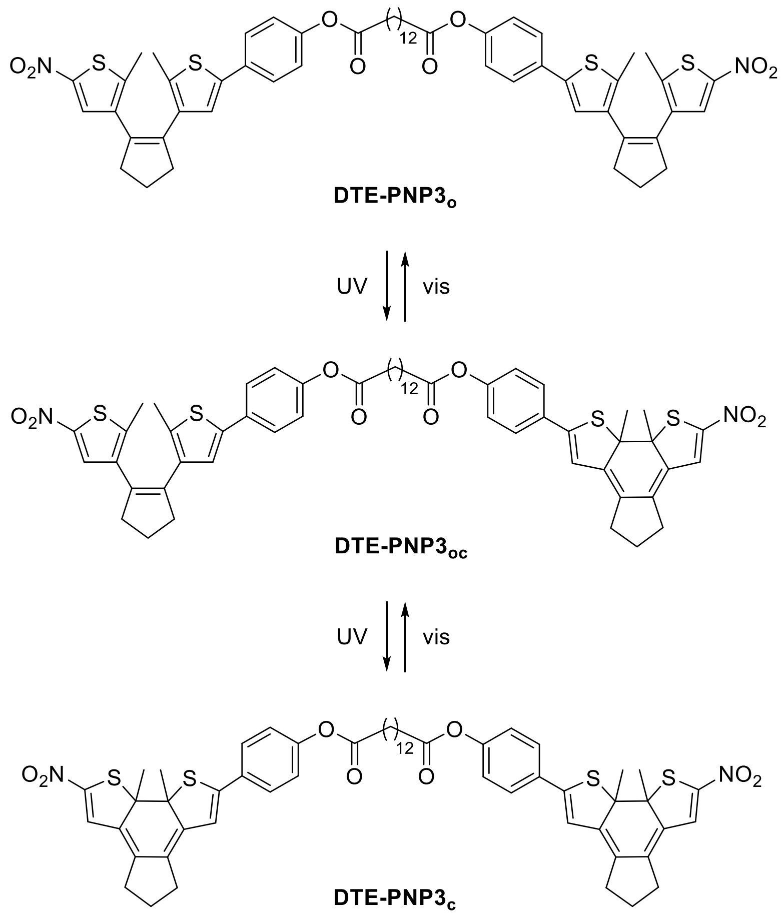

Scheme S2. Photoisomerization process of **DTE-PNP3**. In contrast to **DTE-PNP1** and **DTE-PNP2**, a sequential two-step process is required to toggle from the fully ring-open (**DTE-PNP3$_o$**) to the fully ring-closed state (**DTE-PNP3$_c$**), which proceeds through an intermediate form with one open and one closed DTE units (**DTE-PNP3$_{oc}$**).

Table S3. Photochemical properties of **DTE-PNP3** in $CHCl_3$.[a]

Solvent	$\lambda_{abs,max,o}$ (ε)[b]	$\lambda_{abs,max,c}$ (ε)[b]	$PSS_{o\text{-}c}$[c]	$PSS_{c\text{-}o}$[d]	$\Phi_{o\text{-}c}$[e]	$\Phi_{c\text{-}o}$[f]
**$CHCl_3$**	300 (43.6)	648 (27.6)	0.90	1.00	0.074[g]	0.0064[g]

[a] Photochemical properties were investigated at 365 nm (for photocyclization) and 625/650 nm (for photocycloreversion) based on the UV-vis absorption spectra of the open and closed isomers of **DTE-PNP1**. However, these wavelengths could be further refined by action plot measuring.[8,9] [b] Absorption maximum in nm of the open ($\lambda_{abs,max,o}$) and closed ($\lambda_{abs,max,c}$) isomers. For the closed isomer, the spectral maximum is given for its visible absorption band. The extinction coefficients in 10^3 M^{-1} cm^{-1} are indicated in parenthesis. [c] Molar fraction of closed DTE units after prolonged irradiation at 365 nm, which was determined by ^1H NMR

measurements. Assuming a pure statistical distribution of these units and of the remaining open DTE units, a 1:18:81 ratio can be estimated for **DTE-PNP3$_o$**, **DTE-PNP3$_{oc}$** and **DTE-PNP3$_c$** in the PSS$_{o-c}$ (see Scheme S2). [d] Molar fraction of the open isomer after irradiation at 625 nm determined by UV-vis absorption measurements. [e] Ring-closing quantum yields upon irradiation at 355 nm. [f] Ring-opening quantum yields upon irradiation at 650 nm. [g] Ring-closing and -opening photoisomerization quantum yields were assumed to be the same for the two consecutive photocyclization/photocycloreversion steps required to interconvert between the open and closed states of **DTE-PNP3**.

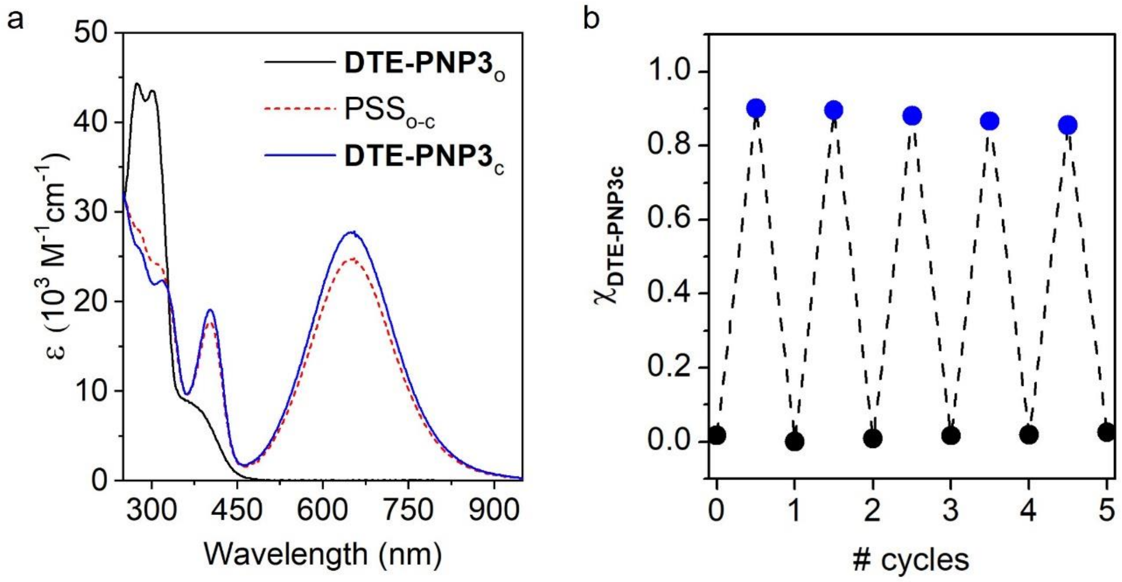

Figure S14. a) **DTE-PNP3** extinction coefficients in $CHCl_3$ for the open isomer **DTE-PNP3$_o$**, the closed isomer **DTE-PNP3$_c$** and the PSS reached under photoexcitation at 365 nm. b) Variation of the molar fraction of the closed DTE units upon consecutive cycles of irradiation of a $CHCl_3$ solution of **DTE-PNP3** at 365 nm (for photocyclization) and 625 nm (for photocycloreversion).

8. Amidation studies with DTE-PNP3 and PNP3

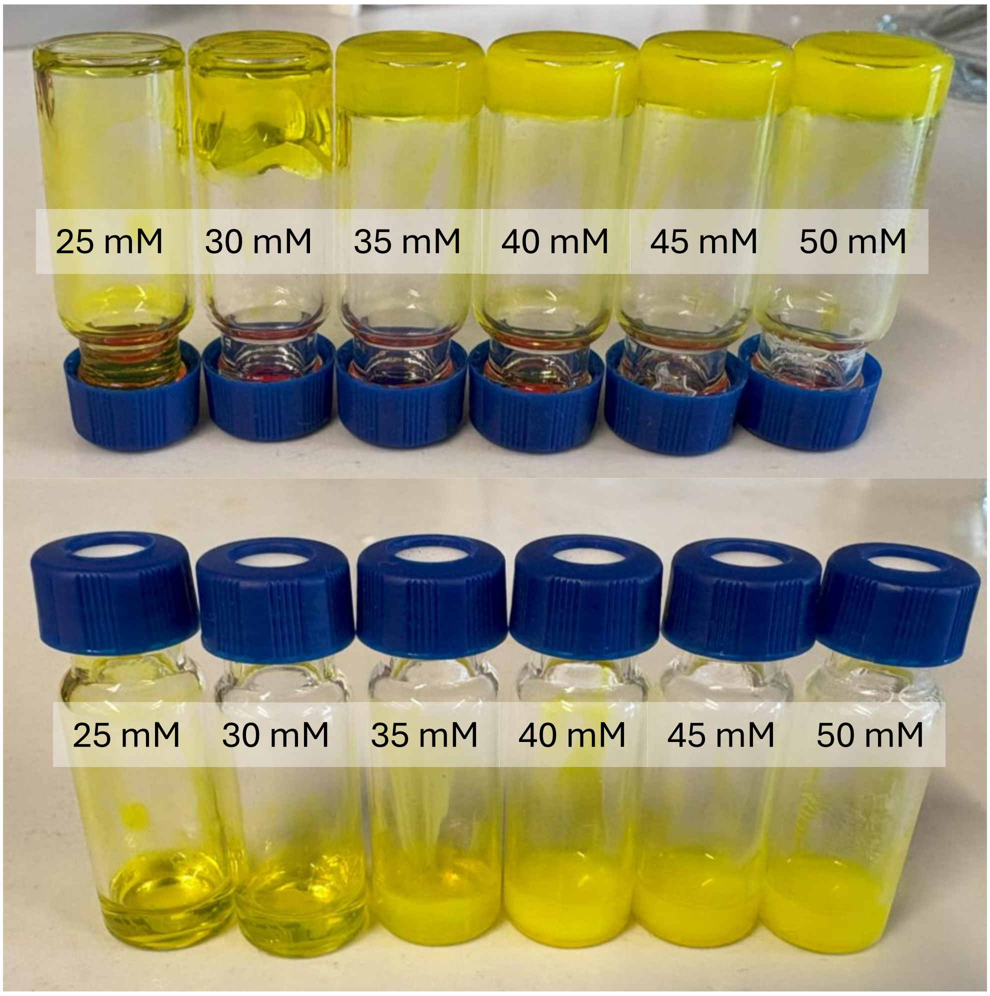

Figure S15. Experiments conducted to optimize the gelation conditions for mixtures of **PEI** (c = 0.3 mM) and **PNP3** (c = 25-50 mM) in $CHCl_3$ via covalent amidation crosslinking. The photographs show the materials obtained for these mixtures after 5 min at room temperature.

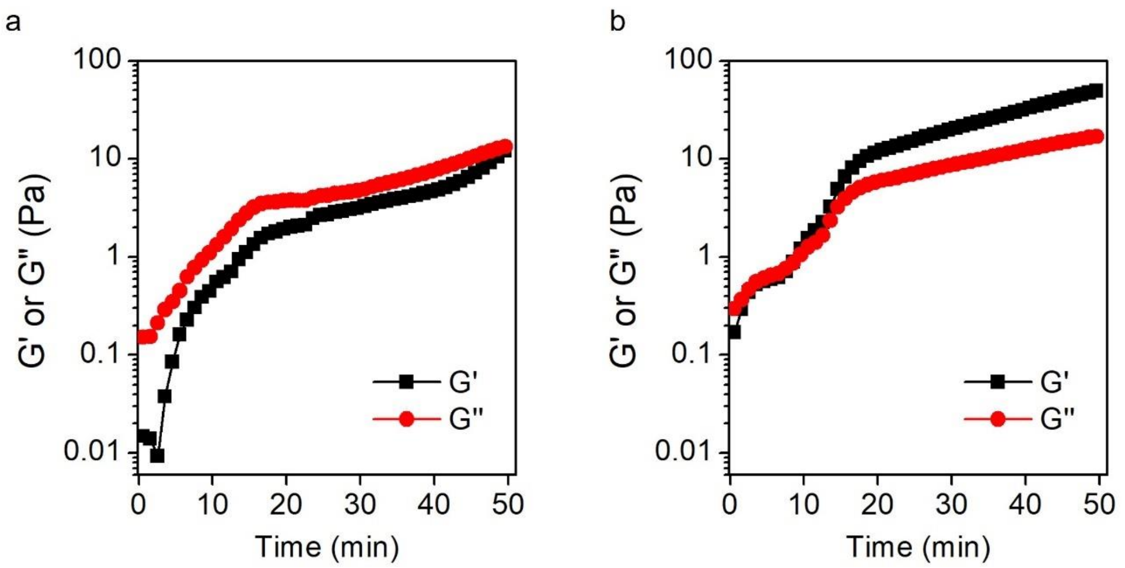

Figure S16. Evolution of the storage (G') and loss (G") moduli of (a) **PEI**:**DTE-PNP3$_o$** and (b) **PEI**:**DTE-PNP3$_c$** solutions in $CHCl_3$ (c_{PEI} = 0.3 mM, $c_{DTE\text{-}PNP3}$ = 2.2 mM). Experiments were conducted at room temperature, with a strain of 1% and a frequency of 1 Hz. For the measurements in (b), **DTE-PNP3** was first irradiated at λ_{max} = 365 nm to generate its closed isomer (*ca*. 90% of closed DTE units).

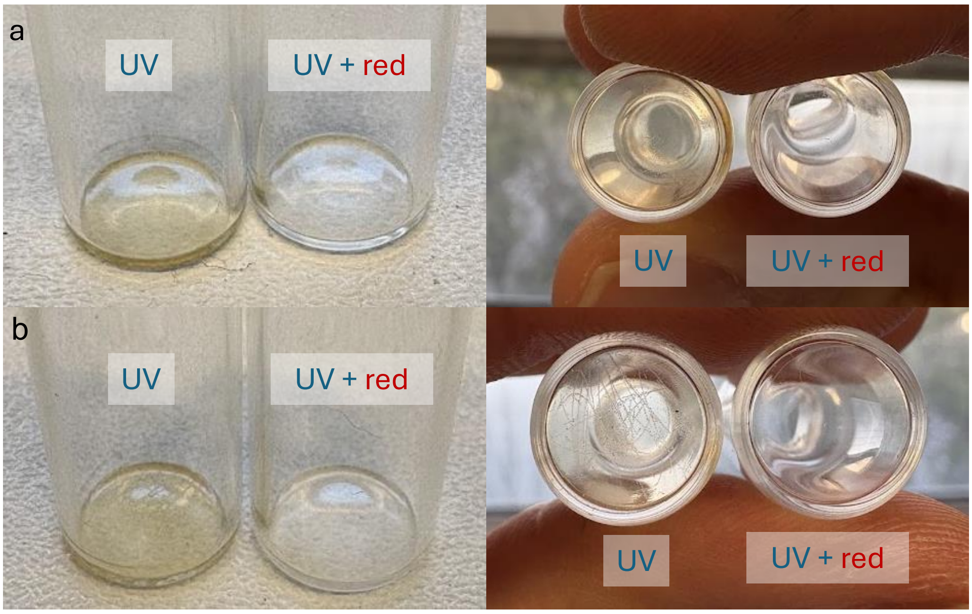

Figure S17. Images of the materials obtained after subjecting 100 µL of a **PEI** (*c* = 0.75 mM) and **DTE-PNP3** (*c* = 10 mM) mixture in $CHCl_3$ film to the following irradiation conditions at room temperature inside a closed vial: UV (365 nm and 3.8 mW cm^{-2}) irradiation for 20 min; and combined UV (365 nm and 3.8 mW cm^{-2}) and red (625 nm and 500 mW cm^{-2}) irradiation. Photographs in (a) show side and bottom views of the irradiated vials after chloroform evaporation in air. Photographs in (b) show side and bottom views of the same samples after scratching the bottom of the vials with a needle, which proves that thin film polymer formation via crosslinking only occurred under sole UV irradiation, while it was inhibited by combined UV and red illumination.

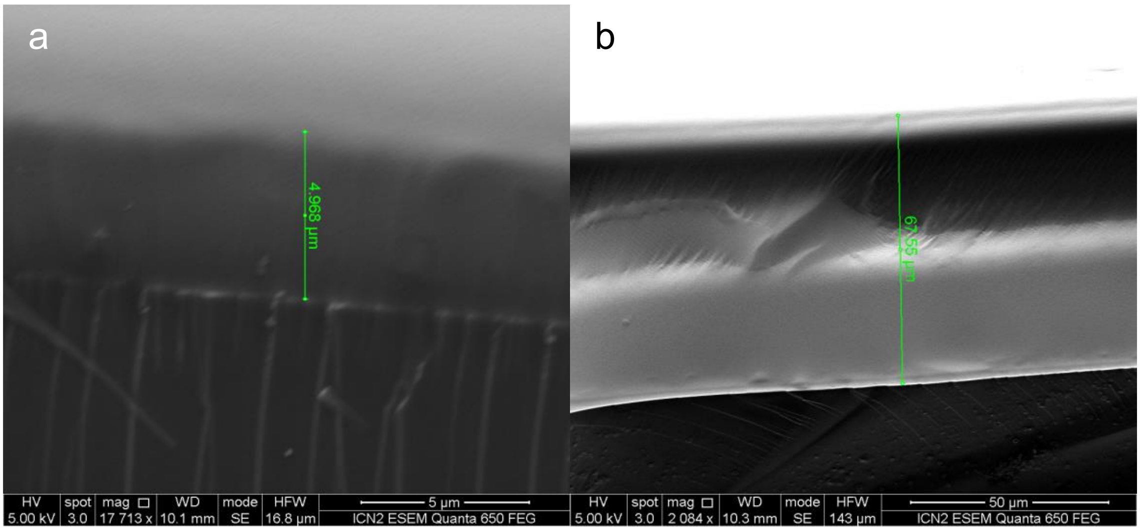

Figure S18. SEM images of the cross-section (at a 70º angle) of the crosslinked polymer films obtained by irradiating a mixture of **PEI** and **DTE-PNP3$_o$** at 365 nm and 3.8 mW cm^{-2} for (a) 15 min and (b) 30 min.

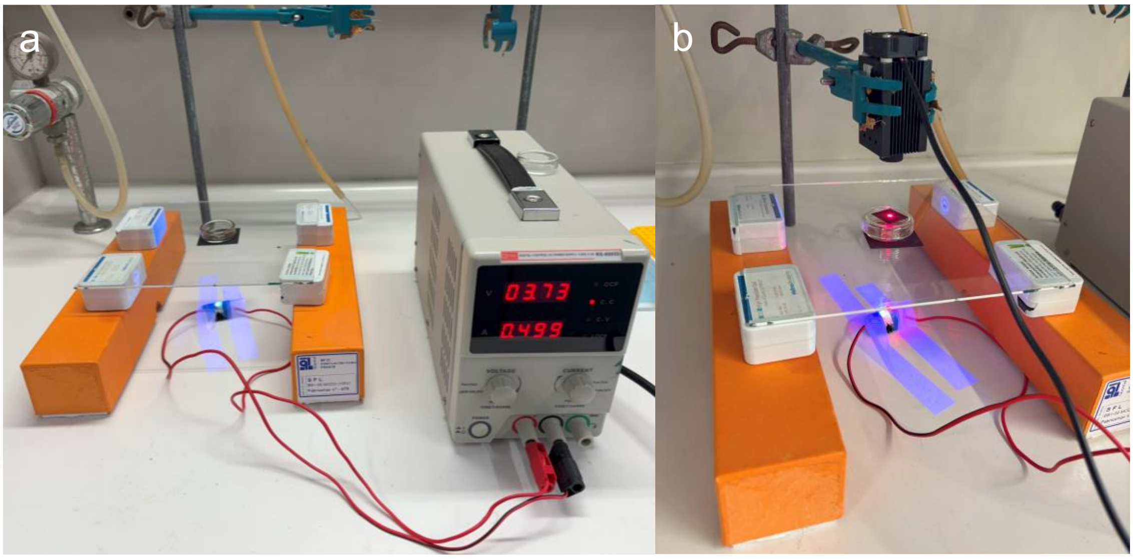

Figure S19. Experimental setups used for the preparation of patterned polymer films under (a) one-color and (b) two-color irradiation. In both cases 600 µL of a $CHCl_3$ solution of **PEI** (*c* = 0.75 mM) and **DTE-PNP3$_o$** (*c* = 10 mM) were poured on a 2 cm-in-diameter glass Petri dish and irradiated from below with a 3W 365 nm LED for the desired time. The UV irradiation power on the sample was selected to be 3.8 mW cm^{-2} by adjusting the sample-LED separation distance and the current applied to the LED. When needed, a mask was placed immediately below the Petri dish to pattern the UV illumination beam. In (b) an additional irradiation source was placed on top of the sample: a 650 nm cw diode laser that was operated at 500 mW cm^{-2}. To pattern the red illumination beam, a black mask was placed on top of the Petri dish lid.

9. NMR and IR spectra of selected compounds

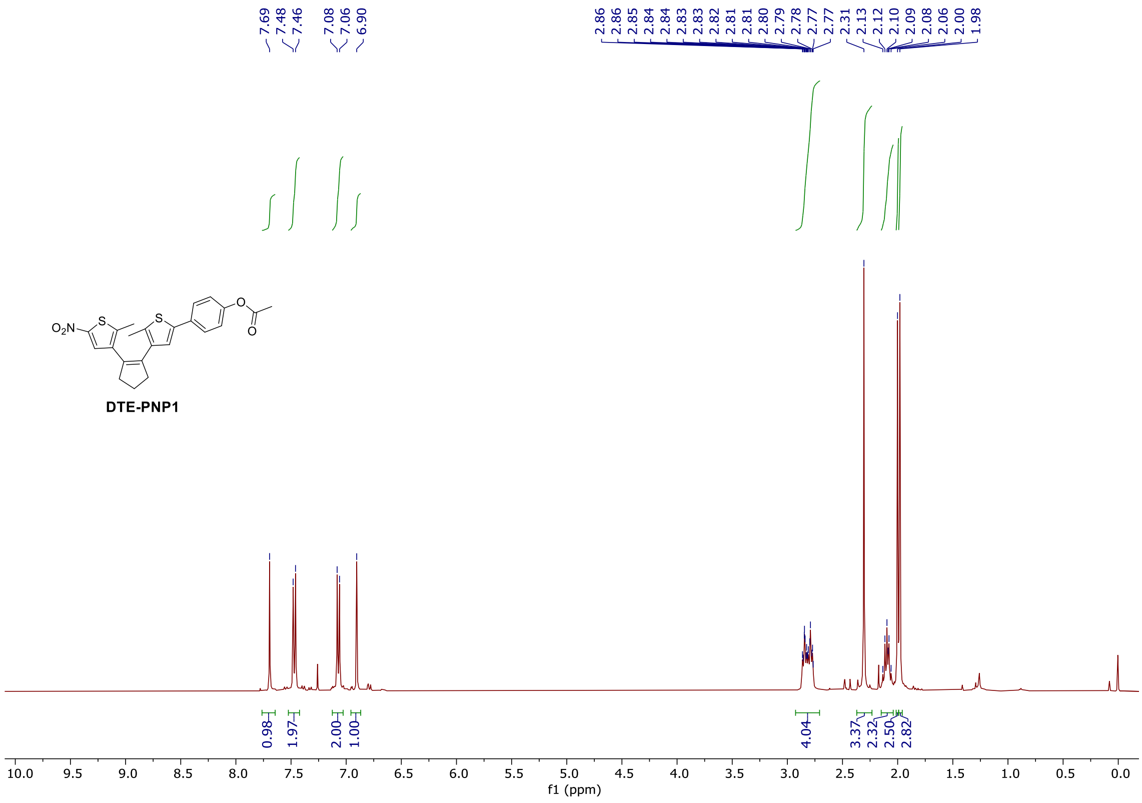

^{1}H NMR (400 MHz, $CDCl_3$)

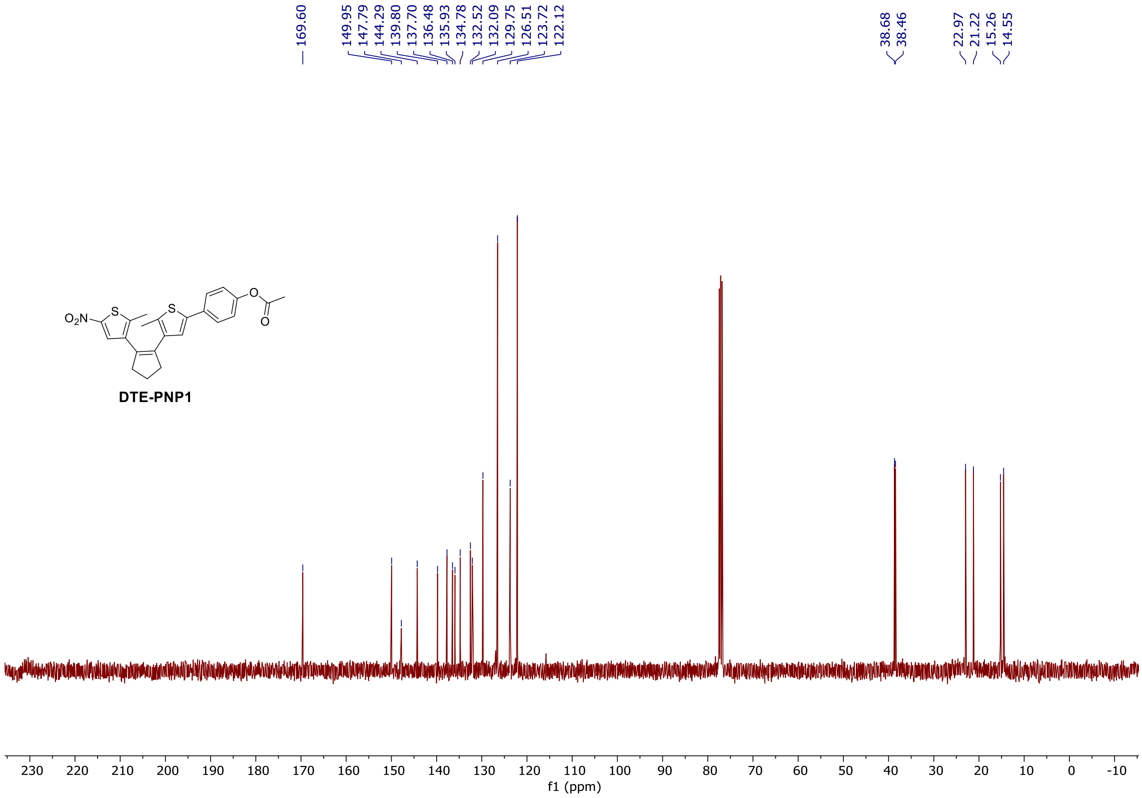

$^{13}C\{^{1}H\}$ **NMR** (101 MHz, $CDCl_3$)

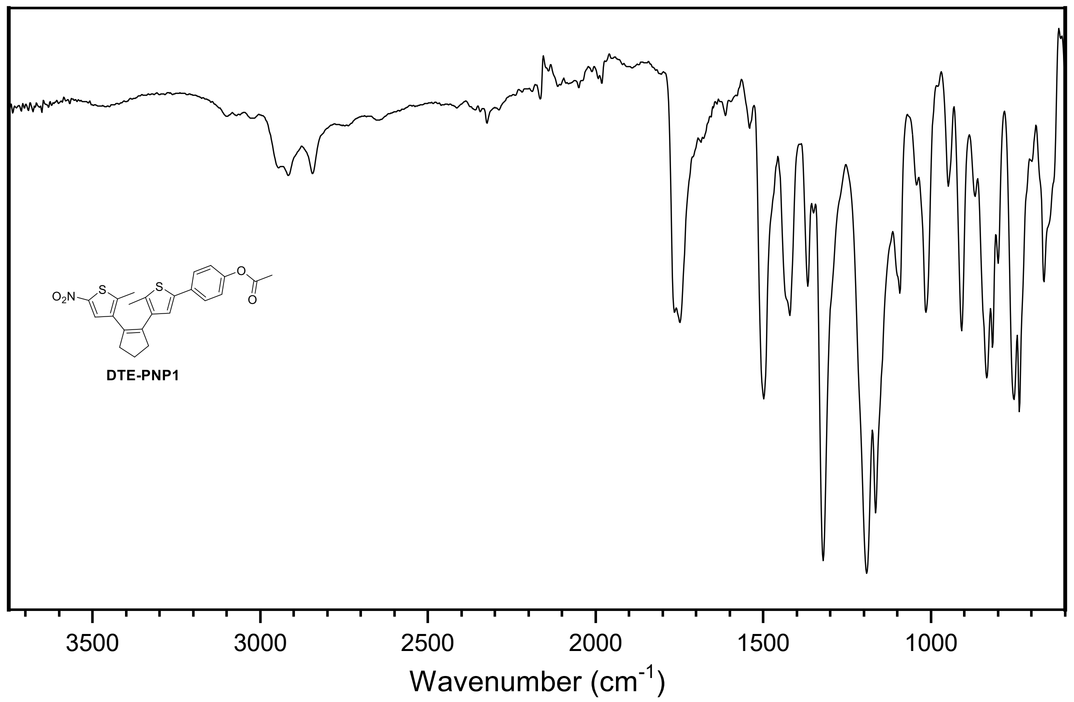

IR (ATR, cm^{-1})

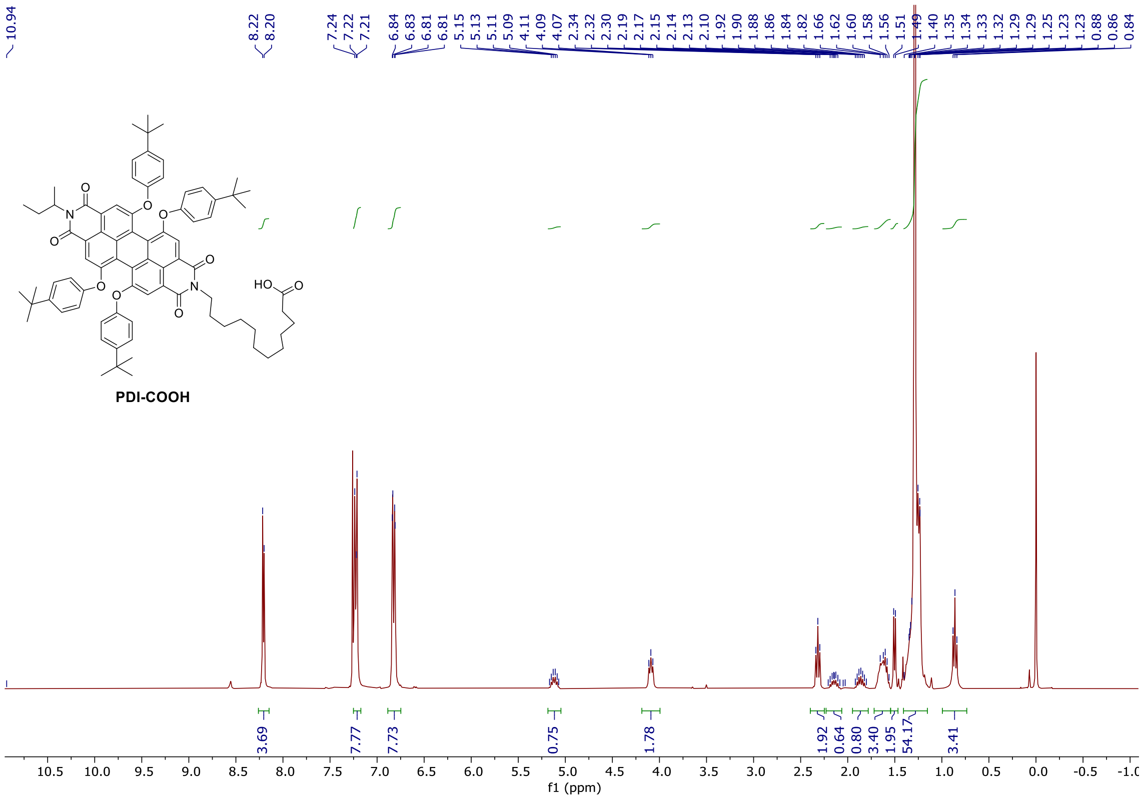

^{1}H NMR (400 MHz, $CDCl_3$)

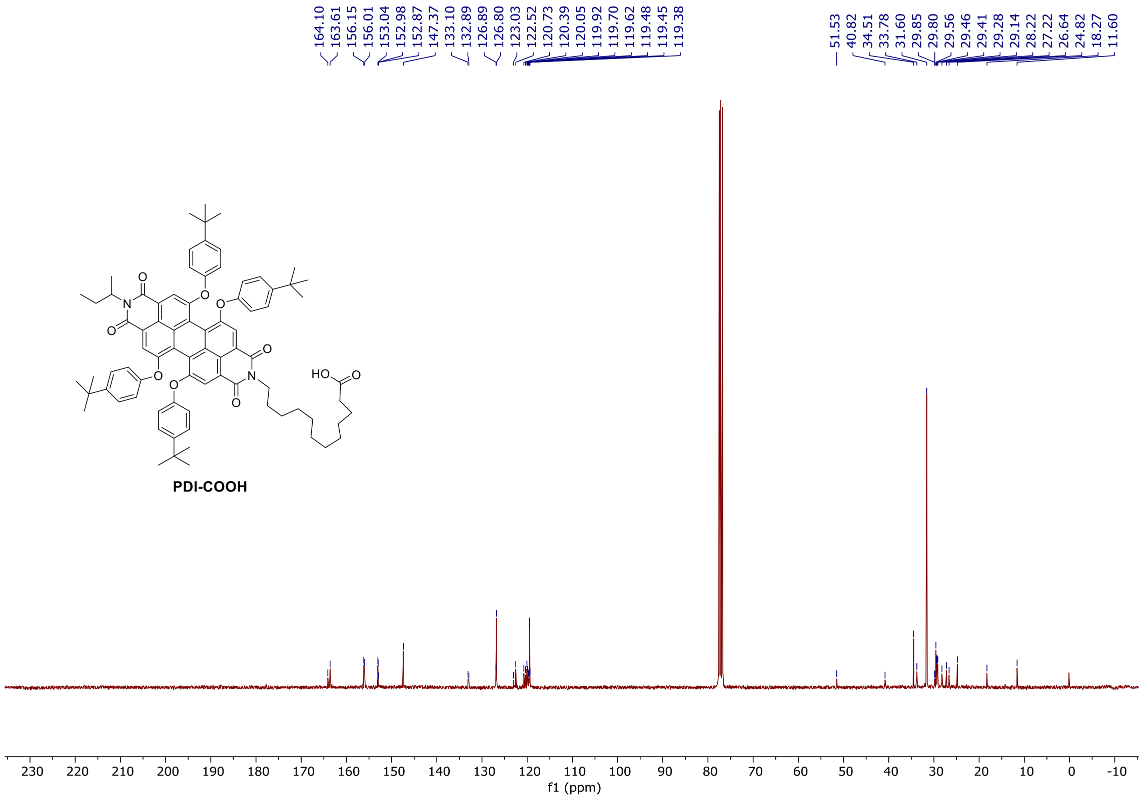

$^{13}C\{^1H\}$ NMR (101 MHz, $CDCl_3$)

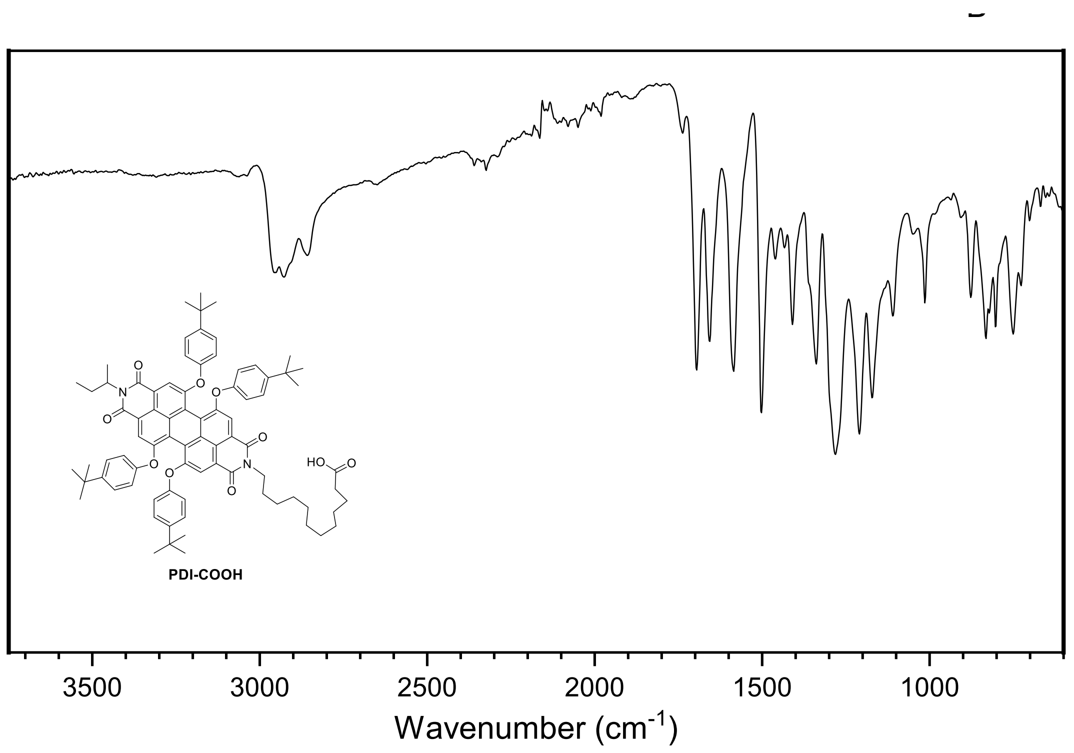

IR (ATR, cm^{-1})

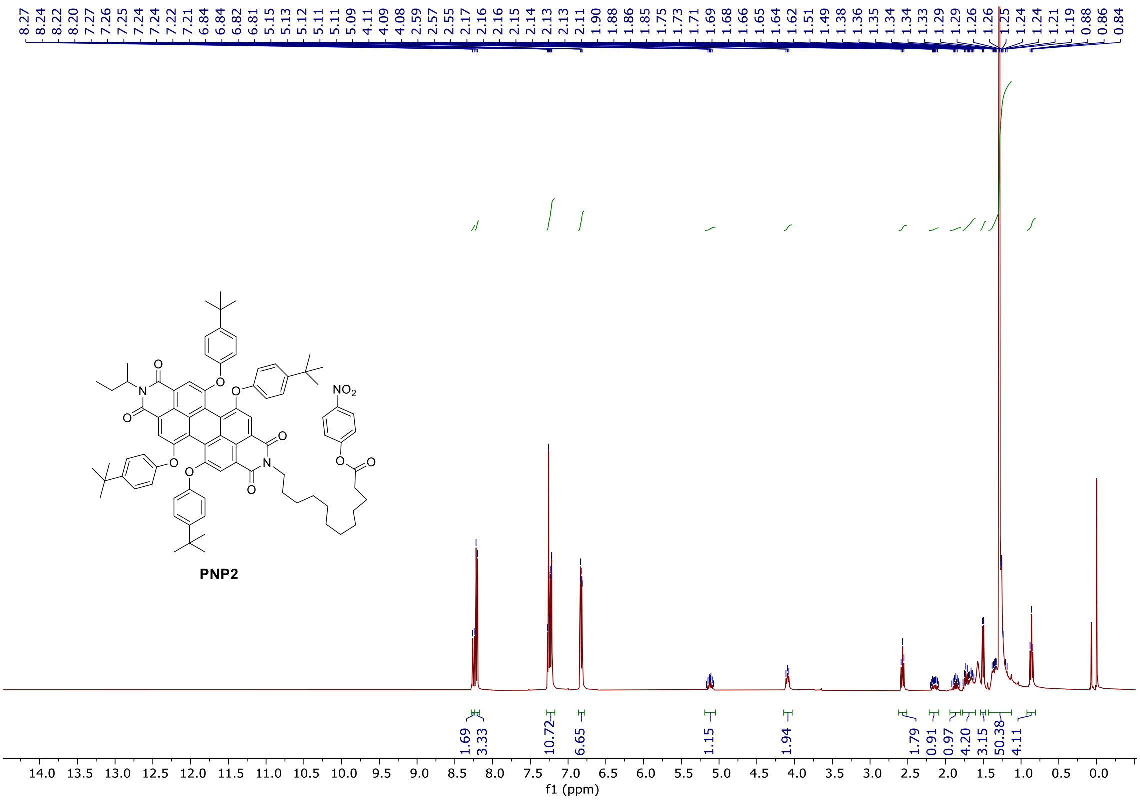

^{1}H NMR (400 MHz, $CDCl_3$)

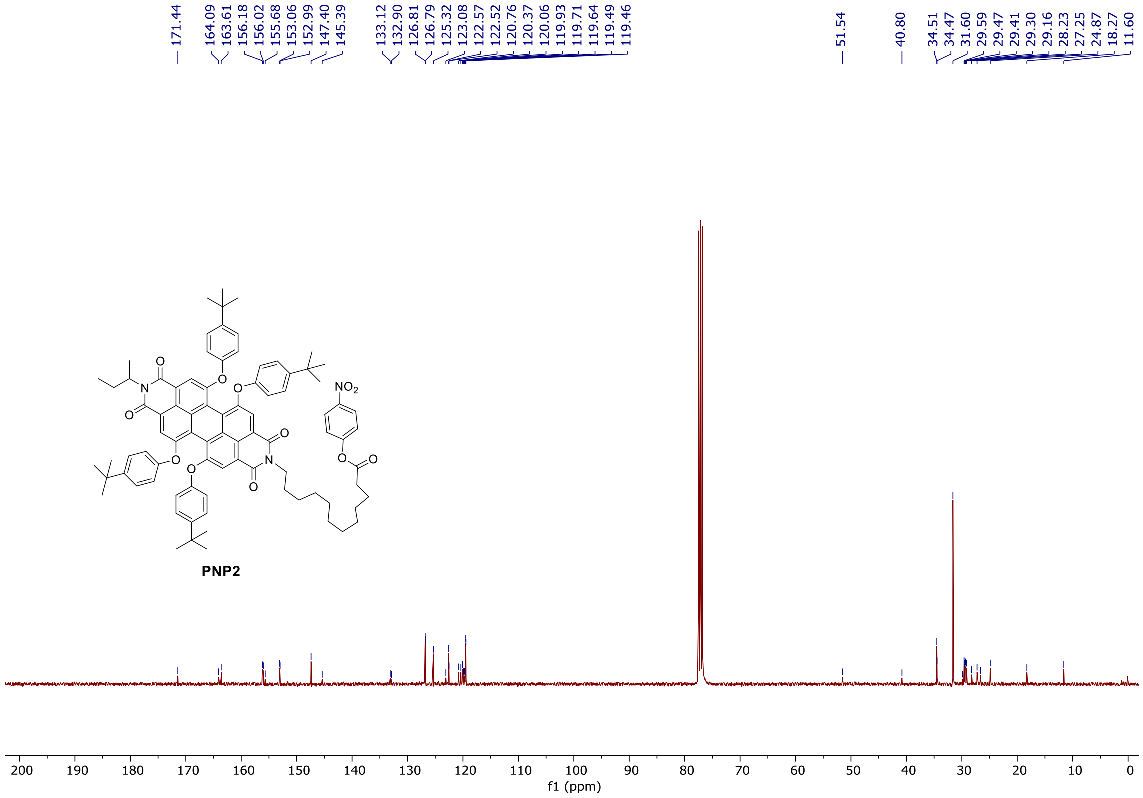

$^{13}C\{^1H\}$ NMR (101 MHz, $CDCl_3$)

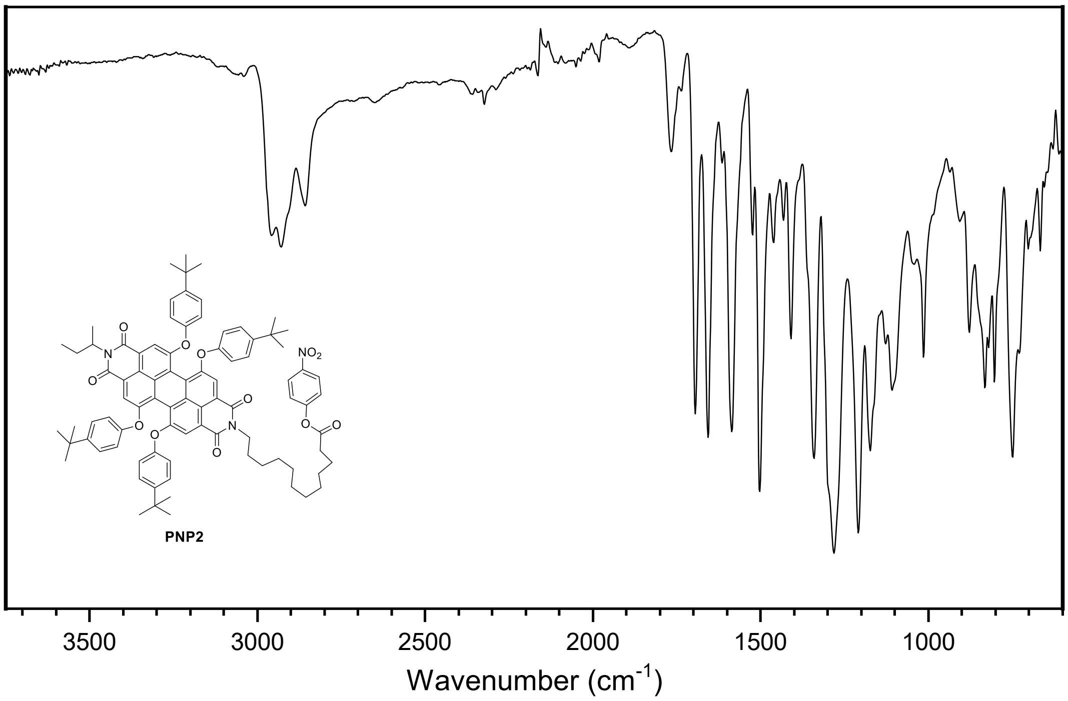

IR (ATR, cm^{-1})

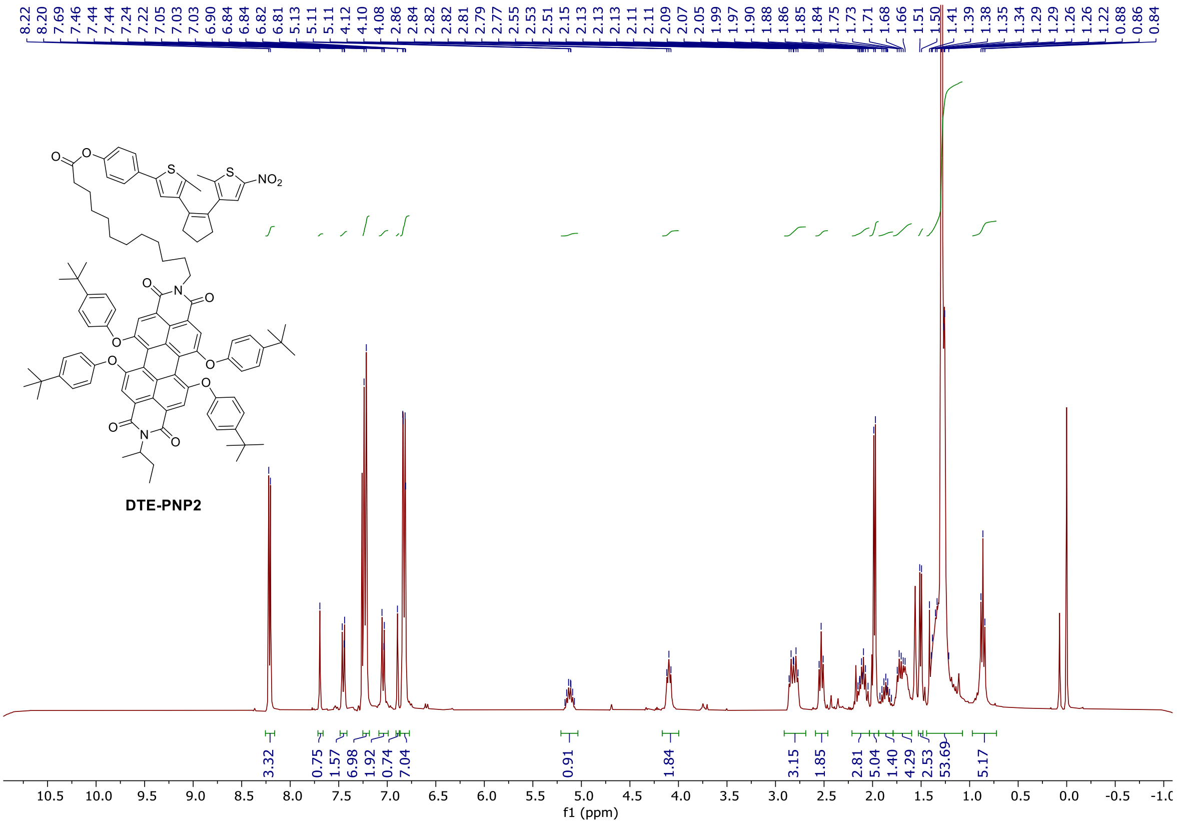

^{1}H NMR (360 MHz, $CDCl_3$)

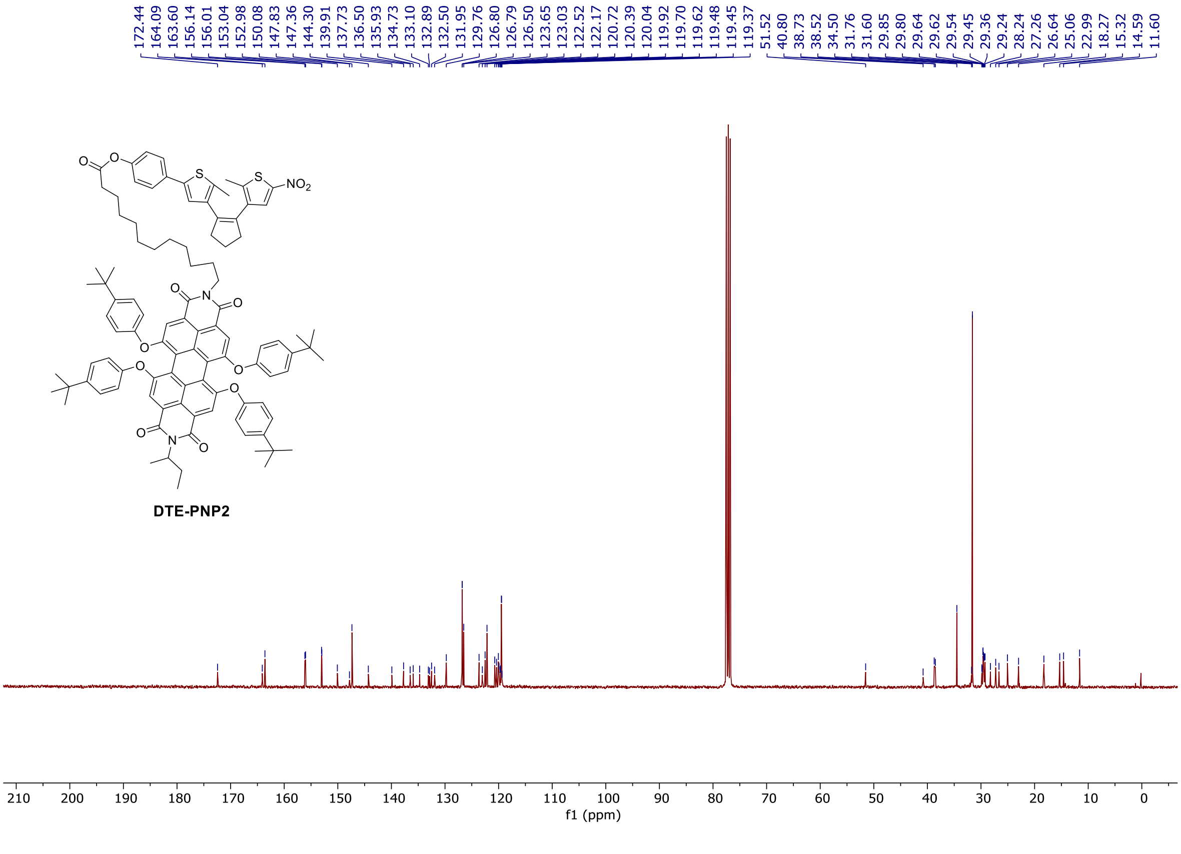

^{13}C{^{1}H} NMR (91 MHz, $CDCl_3$)

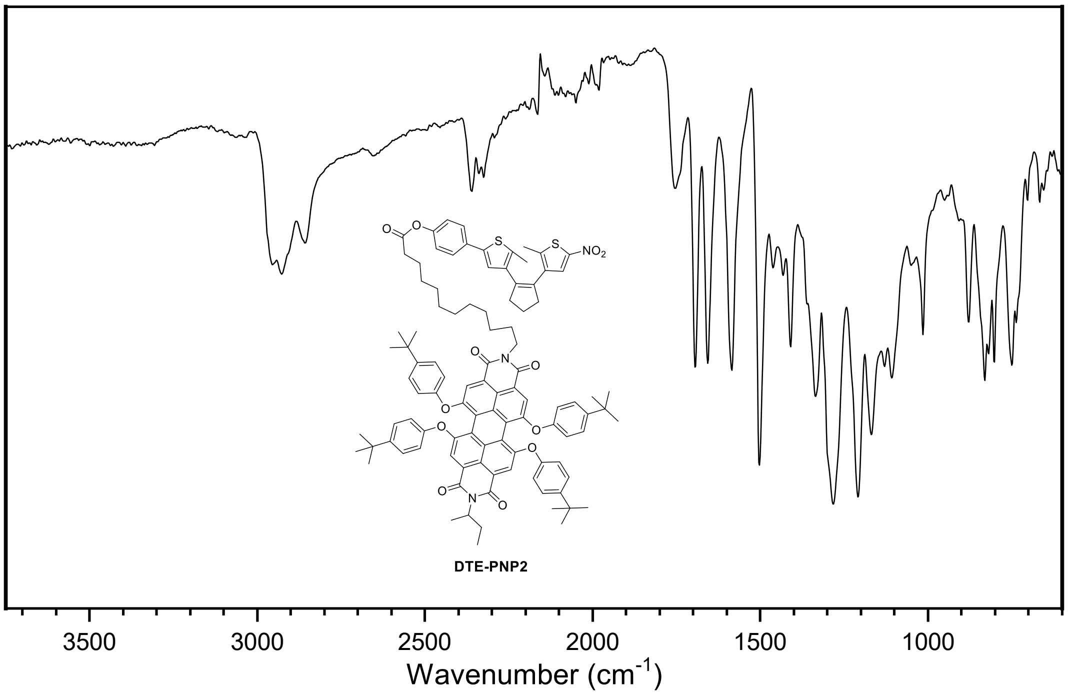

IR (ATR, cm^{-1})

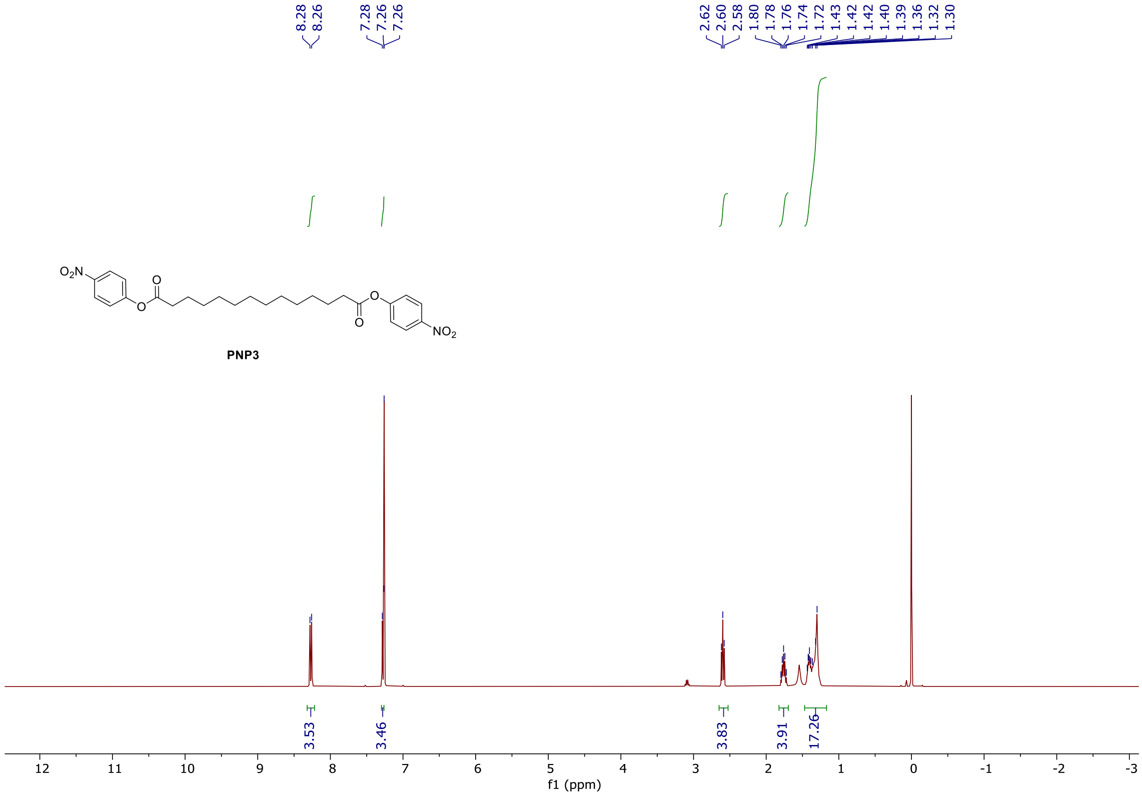

^{1}H NMR (400 MHz, $CDCl_3$)

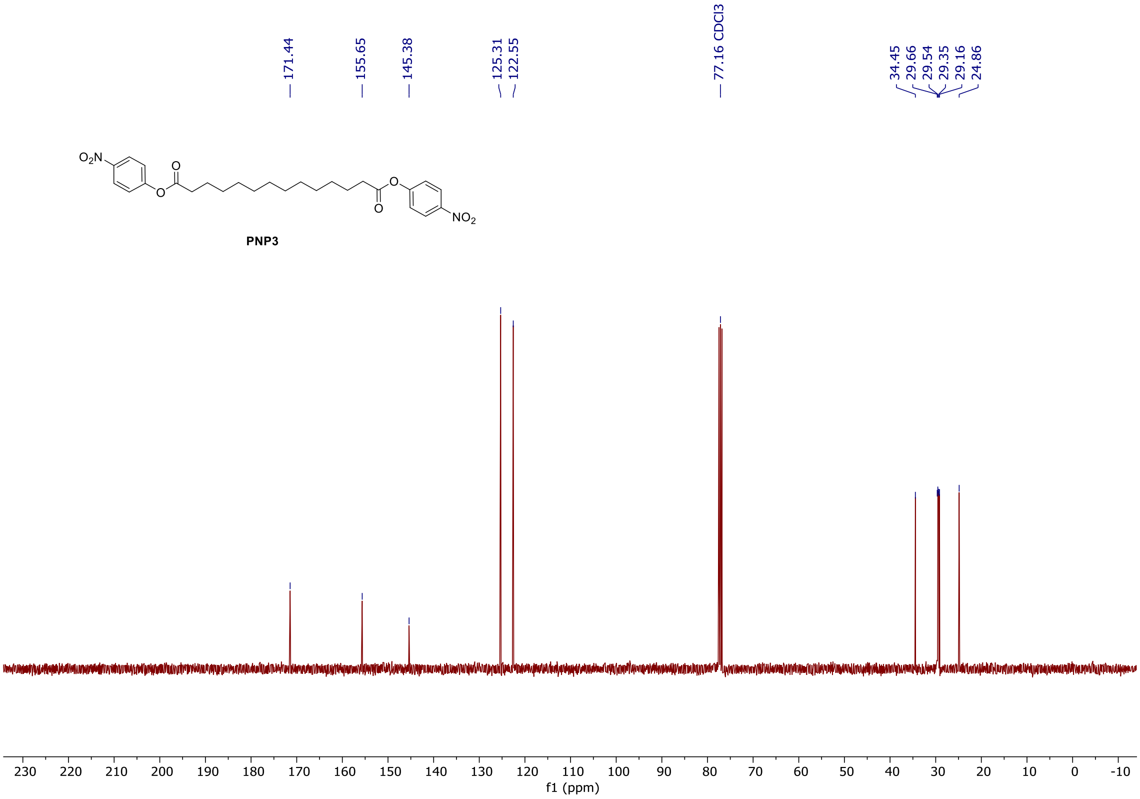

$^{13}C\{^1H\}$ NMR (101 MHz, $CDCl_3$)

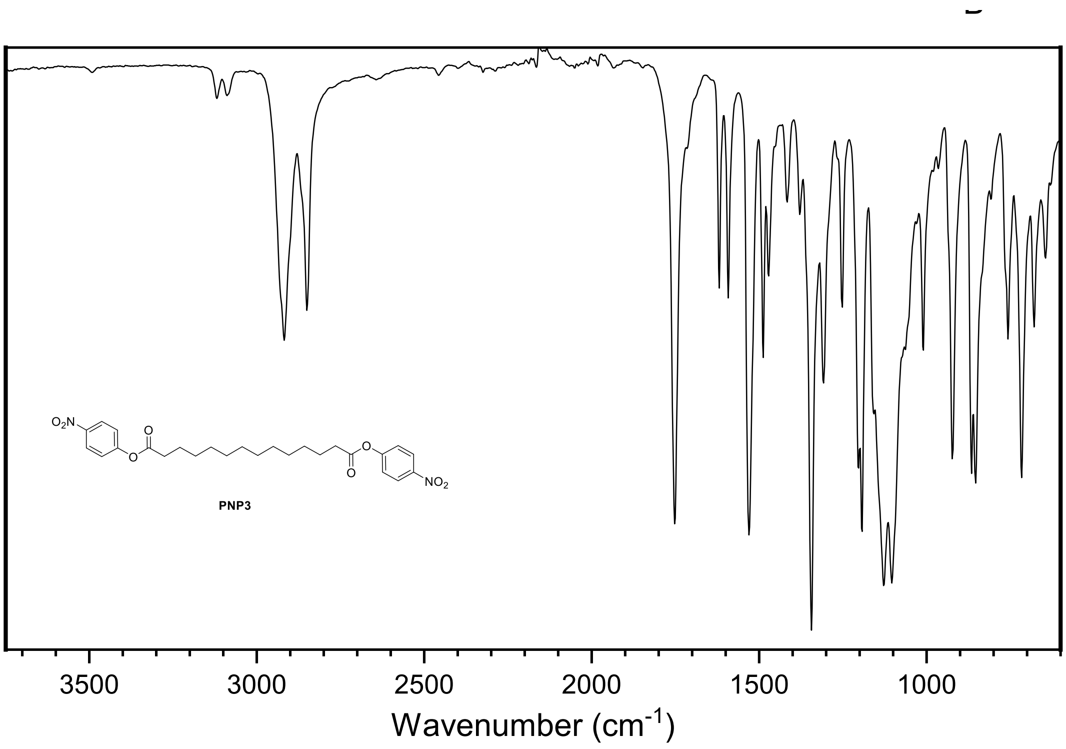

IR (ATR, cm^{-1})

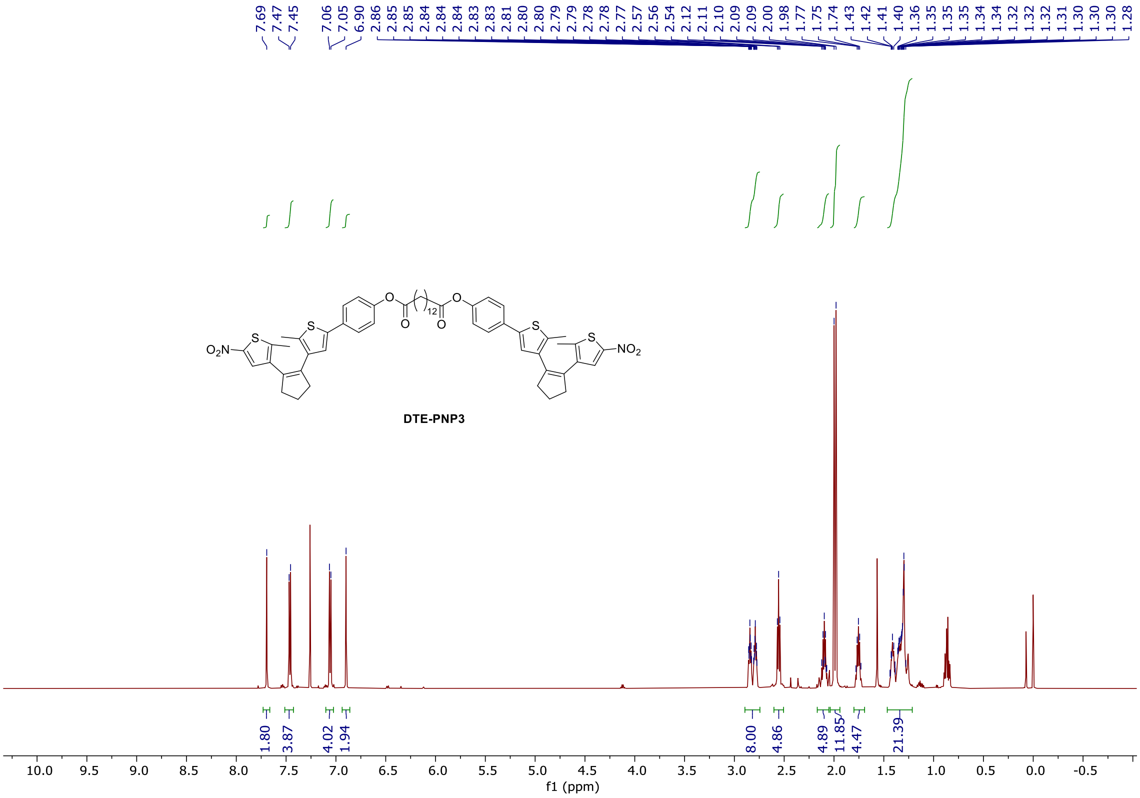

^{1}H NMR (600 MHz, $CDCl_3$)

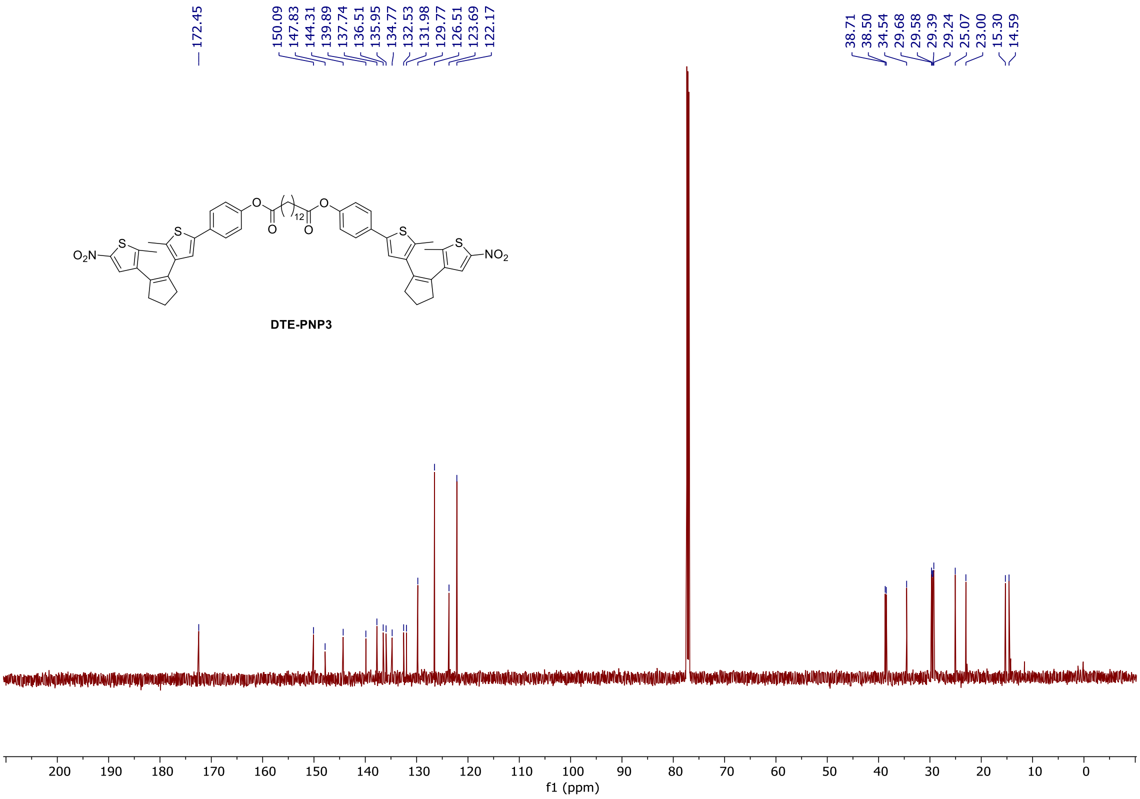

^{13}C{^{1}H} NMR (151 MHz, $CDCl_3$)

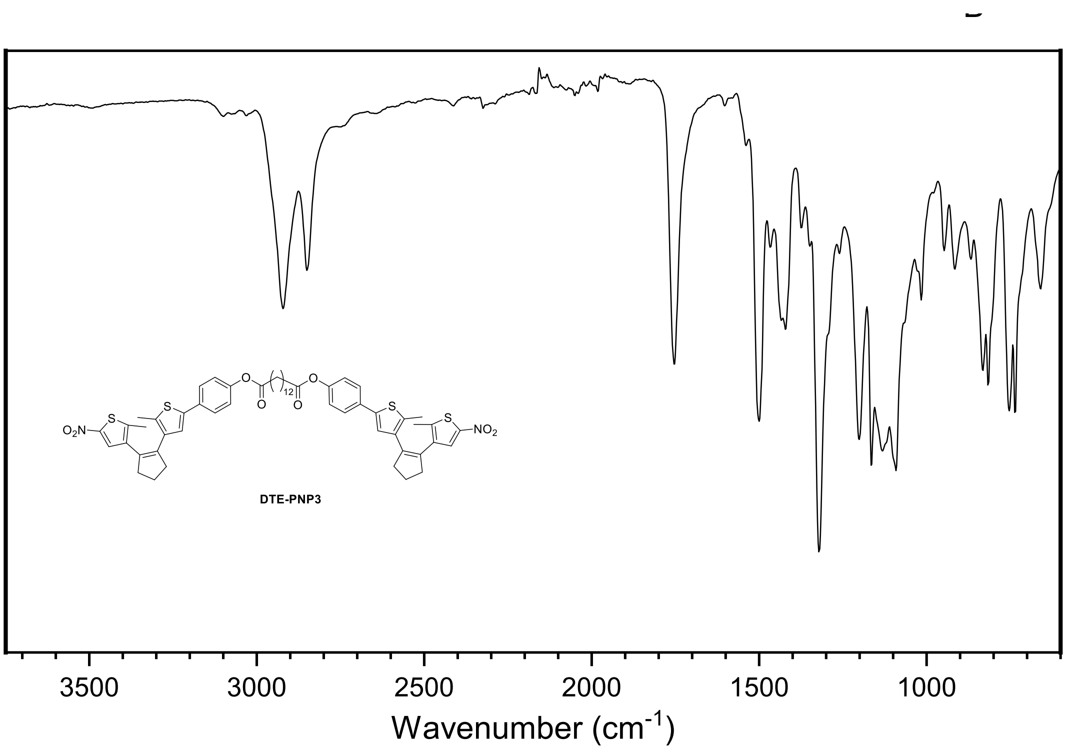
O2N
S
S
O
12
O
O
O
S
S
NO2
DTE-PNP3
3500
3000
2500
2000
1500
1000
Wavenumber (cm-1)

IR (ATR, cm^{-1})

10. References

(1) Lakowicz, J. R. *Principles of Fluorescence Spectroscopy*; 2006. https://doi.org/10.1007/978-0-387-46312-4.

(2) Sánchez, R. S.; Gras-Charles, R.; Bourdelande, J. L.; Guirado, G.; Hernando, J. Light- and Redox-Controlled Fluorescent Switch Based on a Perylenediimide-Dithienylethene Dyad. *J. Phys. Chem. C* **2012**, *116* (12), 7164–7172. https://doi.org/10.1021/jp300815p.

(3) Lees, A. J. A Photochemical Procedure for Determining Reaction Quantum Efficiencies in Systems with Multicomponent Inner Filter Absorbances. *Anal. Chem.* **1996**, *68* (1), 226–229. https://doi.org/10.1021/ac9507653.

(4) Marco, A.; Villabona, M.; Eren, T. N.; Feist, F.; Guirado, G.; Sebastián, R. M.; Hernando, J.; Barner-Kowollik, C. Antagonistic Two-Color Control of Polymer Network Formation. *Adv. Funct. Mater.* **2025**, *35* (7), 2415431. https://doi.org/10.1002/adfm.202415431.

(5) Irie, M.; Lifka, T.; Kobatake, S.; Kato, N. Photochromism of 1,2-Bis(2-Methyl-5-Phenyl-3- Thienyl)Perfluorocyclopentene in a Single-Crystalline Phase. *J. Am. Chem. Soc.* **2000**, *122* (20), 4871–4876. https://doi.org/10.1021/ja993181h.

(6) Troftgruben, M. H. S.; Oldenburg, I.; Pama, A. M.; Igawa, B.; Deguara, S.; Shields, M.; Viswanathan, N. K.; Silverio, J. X.; Gleason, C.; Wang, J. S. M.; Ford, J. S.; Lazaro, H.; Eagon, S. Microwave Mediated Acylation of Alcohols, Phenols and Thiophenols in Neat Acetic Anhydride. *J. Org. Chem.* **2024**, *89* (23), 17850–17852. https://doi.org/10.1021/acs.joc.4c02256.

(7) Villabona, M.; Marco, A.; Sebastián, R. M.; Guirado, G.; Hernando, J. Amplified Light-Induced PKa Modulation with Diarylethene Photoswitches. *J. Org. Chem.* **2024**, *89* (24), 17991–18002. https://doi.org/10.1021/acs.joc.4c01606.

(8) Irshadeen, I. M.; Walden, S. L.; Wegener, M.; Truong, V. X.; Frisch, H.; Blinco, J. P.; Barner-Kowollik, C. Action Plots in Action: In-Depth Insights into Photochemical Reactivity. *J. Am. Chem. Soc.* **2021**, *143* (50), 21113–21126. https://doi.org/10.1021/jacs.1c09419.

(9) Walden, S. L.; Carroll, J. A.; Unterreiner, A.-N.; Barner-Kowollik, C. Photochemical Action Plots Reveal the Fundamental Mismatch Between Absorptivity and Photochemical Reactivity. *Adv. Sci.* **2024**, *11* (3), 2306014. https://doi.org/10.1002/advs.202306014

.
